# Altered oligodendroglial cell development and increased myelination in *SCN2A* gain-of-function-linked epilepsy

**DOI:** 10.1186/s40478-026-02437-1

**Published:** 2026-10-07

**Authors:** Constanza Bravo-Rossainz Baez, Xian Xin, Hang Lyu, Daniil Kirianov, Ying Sun, Erik Späte, Mariapia Grassia, Birgit Engeland, Malte Stockebrand, Konstantin Khodosevich, Ulrike B. S. Hedrich, Dirk Isbrandt, Friederike Pfeiffer

**Affiliations:** 1 Department of Neurophysiology, Institute of Physiology, University of Tübingen, Tübingen, Germany; 2 Biotech Research and Innovation Centre (BRIC), Faculty of Health and Medical Sciences, University of Copenhagen, Copenhagen, Denmark; 3 Department of Neurology and Epileptology, Hertie Institute for Clinical Brain Research, University of Tuebingen, Tübingen, Germany; 4 Deutsches Zentrum Für Neurodegenerative Erkrankungen (DZNE), Bonn, Germany; 5 Institute for Molecular and Behavioral Neuroscience and Center for Molecular Medicine Cologne, University of Cologne, Cologne, Germany; 6 Werner Reichardt Centre for Integrative Neuroscience, University of Tübingen, Tübingen, Germany

## Abstract

Gain-of-function variants in *SCN2A,* encoding the voltage-gated sodium channel Na_V_1.2, cause developmental and epileptic encephalopathies characterized by seizures, developmental delay, and white matter abnormalities. While Na_V_1.2 is known to regulate neuronal excitability, its role in oligodendroglial cells is unclear. Here, we show that Na_V_1.2 is expressed in early oligodendrocyte lineage stages. In mice carrying the gain-of-function *Scn2a*^*p.A263V*^ variant, oligodendrocyte precursor cells displayed similar Na_V_1.2 expression, but altered electrophysiological properties and calcium signaling. Single-cell transcriptomics revealed stage-specific changes in oligodendroglial maturation during early postnatal development, associated with lower variability and slightly faster conduction velocities. In adult mutant mice, gray matter myelination was increased without changes in oligodendrocyte numbers or myelin structure. Since Na_V_1.2 was absent from mature oligodendrocytes, the enhanced myelination likely reflects an activity-dependent adaptation to altered neuronal network activity rather than a primary myelinopathy. These findings identify a glial contribution to *SCN2A*-related disease mechanisms and underscore the need to consider neuron–glia interactions when developing targeted therapies for epilepsy.

**Supplementary Information:**

The online version contains supplementary material available at https://doi.org/10.1186/s40478-026-02437-1.

## Introduction

Approximately one quarter of inherited epilepsy syndromes are associated with genetic variants in ion channel genes [51, 67], including *SCN2A*, which encodes the voltage-gated sodium channel Na_V_1.2 [37]. Na_V_1.2 is expressed during human and mouse brain development and is essential for the initiation and propagation of action potentials in neurons. Variants in *SCN2A* are associated with a spectrum of neurological disorders, including infantile epileptic encephalopathy (IEE), self-limited familial neonatal-infantile epilepsy (SeLFNIE), and autism spectrum disorder/intellectual disability (ASD/ID) [5, 76]. These variants can lead to either gain- or loss-of-function effects, with partially overlapping phenotypic spectra. Gain-of-function variants can cause a broad spectrum of neonatal- or infantile-onset epilepsies, ranging from mild self-limited phenotypes to severe developmental and epileptic encephalopathies (DEEs), whereas loss-of-function variants are associated with neurodevelopmental disorders and can also cause infantile- or childhood-onset DEEs [4, 75, 92]. The effects of dysregulated sodium channel activity, including that of Na_V_1.2, on the channels’ biophysical properties and intrinsic properties of neurons as critical drivers of circuit pathology, have been well studied [4, 22, 37, 48, 85, 91–93]. In contrast, the effects on non-neuronal brain cells in channelopathies are less well characterized. Magnetic resonance imaging (MRI) and neuropathological studies in neonates and adults with epilepsy [36, 77] and in patients with DEEs are often linked to pathogenic ion channel variants, frequently revealing white matter abnormalities including delayed myelination, reduced myelin volume, and widespread white matter signal abnormalities—often with region-specific patterns [1]. Concerning Na_V_1.2, the oligodendrocyte-specific knockout of *Scn2a* in mice caused myelination deficits, reduced myelin thickness, impaired circuit function in the auditory brainstem, and resulted in sensory hypersensitivity mimicking autism spectrum symptoms [2].

Myelin is a dynamic structure that is formed during development and can be remodeled throughout life [13, 39, 61]. Neuronal activity induces myelination [20, 59] and myelin remodeling [25, 33, 58]. Dysregulated myelination, as has been addressed in white matter disorders such as multiple sclerosis (MS), results in impaired neural communication and neurodegeneration [29].

Oligodendrocyte precursor cells (OPCs) are a dynamic and persistent cell population, comprising approximately 5–10% of all brain cells [14, 17, 70]. Originating early in development, OPCs retain the capacity to proliferate and differentiate into myelinating oligodendrocytes throughout life [63]. Oligodendrocytes are responsible for the formation of myelin, which enables the rapid propagation of action potentials along axons via saltatory conduction. Notably, axonal electrical activity has been shown to influence OPC behavior in return. Thereby, synaptic inputs, including glutamatergic and GABAergic signals [7], modulate OPC proliferation and differentiation [15, 33].

In humans, the recurrent *SCN2A* p.(Ala263Val) (A263V) variant causes a gain-of-function of Na_V_1.2 and is associated with a broad clinical spectrum of variable severity, including SeLFNIE and childhood-onset episodic ataxia, severe ID, early infantile epileptic encephalopathy (EIEE, Ohtahara syndrome), intractable neonatal-onset epileptic encephalopathy, and increased mortality [54, 92]. When introduced into mice, the variant causes a gene-dose-dependent neuronal hyperexcitability and spontaneous epilepsy phenotype, with frequent neonatal hippocampal seizures, but a self-limiting phenotype in the heterozygous condition [74]. In adult mice, however, the presence of the *SCN2A* (p.Ala263Val) allele exacerbated the disease course in the experimental autoimmune encephalomyelitis (EAE) mouse model of multiple sclerosis [78].

In addition to the prominent expression in neurons, sequencing studies detected RNA encoding Na_V_1.2 also in oligodendroglial cells [18, 56, 82, 88]. Sodium currents have been measured in oligodendroglial cells. However, their presence depends on the animal's age and the cell's state [41]. Na_V_1.2 expression in pre-myelinating oligodendrocytes has been associated with their differentiation, and deletion of *Scn2a* has been shown to impair maturation of a specific subset of pre-myelinating OLs [8, 34], but the consequences of gain-of-function *SCN2A* variants for oligodendroglial cell development and function have not been studied to date.

Here, we characterized *Scn2a* expression in oligodendroglial cells across developmental stages using single-cell RNA sequencing (scRNA-seq) and assessed Na_V_1.2 protein levels by immunocytochemistry, comparing the expression levels of the *Scn2a*^*p.A263V*^ variant in heterozygous mutant mice with controls. We further investigated whether the *Scn2a*^*p.A263V*^ variant affects the development and physiology of oligodendroglial cells as well as overall myelination in the mouse brain. Because we did not detect Na_V_1.2 in mature oligodendrocytes, we suggest that the overall increase in myelination in the gray matter of *Scn2a*^*p.A263V*^ mutant mice was a consequence of altered neuronal activity rather than a primary change in oligodendrocyte function. Our findings provide insights into the importance of Na_V_1.2 in regulating oligodendroglial lineage cells in *SCN2A*-linked developmental epilepsies.

## Methods

### Animals

Heterozygous Tg(Cspg4-DsRed.T1)1Akik/J (strain #008241) were crossed with heterozygous *Scn2a*^*p.A263V*/+^ mice, originally described by Schattling et al. [78].

Mice were housed in a controlled environment under a 12-h light–dark cycle.

All experiments were performed in accordance with European, national (the German Animal Welfare Law) and institutional guidelines and the approval by the (local) authorities (Regierungspräsidium Tübingen) and the State Office of North Rhine-Westphalia, Department of Nature, Environment and Consumer Protection (LANUV/LAVE NRW, Germany).

### Tissue processing

Animals were perfused on day 15 (P15) or day 60 (P60) and fixed with a 4% paraformaldehyde (PFA) solution (Roti Histofix, Carl Roth, No. 5666.2). Brains were dissected, post-fixed overnight in 4% PFA, and washed 5 times in PBS. Following incubation in 25% sucrose in PBS for at least 48 h, brains were embedded in O.C.T. compound (Tissue-Tek® O.C.T, Sakura, #4583) and stored at − 80 °C. Coronal brain slices (70 μm thickness) were obtained using a cryostat (Leica CM1950, Leica Microsystems, Germany) and immediately transferred to PBS. For the analysis of oligodendroglial cells in white matter at P10, P20, and P50, murine brain slices of a thickness of 400 µm were generated with a Leica VT 1200S vibratome using the following solution: 87 mM NaCl, 2.5 mM KCl, 1.25 mM NaH2PO4*H2O, 7 mM MgCl2, 0.5 mM CaCl2, 25 mM NaHCO3, 25 mM glucose, 75 mM sucrose. Slices were fixed overnight in 4% PFA at 4 °C, embedded in agar, re-sectioned to 35-µm-thick slices, and stored in PBS until further use.

### Culture of primary oligodendrocyte precursor cells (OPCs)

For OPC cultures, C57BL/6N, DsRed^+/-^ x heterozygous *Scn2a*^*p.A263V*/+^ or DsRed^+/-^ x *Scn2a*^+*/*+^ pups were used. Primary OPCs were isolated following an adapted protocol from Freeman et al. [100] and optimized for efficient cultivation from murine brain tissue. Briefly, wild-type and mutant mice (P0–P4) were sacrificed by decapitation. Litters of three to four animals were processed concurrently. Following decapitation, brains were carefully dissected, and non-cortical regions, including the meninges, hippocampus, cerebellum, and midbrain, were removed to isolate cortical tissue (Table 1).

**Table 1:** Composition of OPC media used

	Concentration	Manufacturer
*DMEM-Sato Base Growth Medium for OPCs*		
DMEM high glucose (11,960–044)		Gibco
N2 supplement (N2-K)	1X	Capricorn Scientific
L-Glutamine (25,030–081)	1 mM	Gibco
Pen/Strep (15,140–122)	100U/ml	Gibco
Sodium Pyruvate (11,360–070)	1 mM	Gibco
N-acetyl-L-cysteine /A8199)	5 µg/ml	Sigma
Trace elements B	1X	Corning
d-Biotin (B4639)	10 ng/ml	Sigma
*Proliferation Medium (add to DMEM-Sato Base Growth Medium)*		
NT3 (450–03)	1 ng/ml	PeproTech
PDGF AA (100-13A)	10 ng/ml	PeproTech
*Differentiation Medium (add to DMEM-Sato Base Growth Medium)*		
T3	400 ng/ml	Sigma
T4	400 ng/ml	Sigma
Forskolin (F6886)	4.2 ng/ml	Sigma
CNTF (450–50)	10 ng/ml	PeproTech
*Myelination Medium*		
DMEM high glucose (11,960–044)		Gibco
N2 supplement (N2-K)	1X	Capricorn Scientific
T3	40 ng/ml	Sigma
T4	40 ng/ml	Sigma
d-Biotin (B4639)	10 ng/ml	Sigma
Vitamin B12	27.2 ng/ml	Millipore
Ceruloplasmin	100 ng/ml	Millipore
Hydrocortisone	0.05 µM	Millipore
CNTF (450–50)	0.1 ng/ml	PeproTech
Sodium Pyruvate (11,360–070)	1 mM	Gibco

Cortices were enzymatically digested in 2.5% trypsin solution and mechanically dissociated to obtain a single-cell suspension. OPCs were then selectively isolated using immunopanning as described by Emery & Dugas [23]. The cell suspension was then subjected to sequential immunopanning by incubation in antibody-coated dishes, beginning with negative selection (bovine serum albumin solution), followed by positive selection with antibodies against A2B5 and PDGFRα to enrich for oligodendrocyte precursor cells (OPCs) selectively. For wild-type mice (C57BL/6), cortices from multiple animals were pooled prior to immunopanning. In contrast, tissue from mutant mice was processed individually because the genotype was initially unknown, thus ensuring accurate OPC isolation.

Isolated cells were plated at a density of 20,000 cells per well in four-well LabTek chambers (Thermo Fisher Scientific), pre-coated with Poly-L-Lysine hydrobromide (Sigma, 25,988–63-0) to enhance adhesion and growth. Cultures were maintained at 37 °C with 5% CO_2_ in a carbon dioxide incubator (HERAcell 150i, Thermo Fisher Scientific). Cultures were maintained in proliferation medium, with medium changes on day 2. On this day, some wells continued to be maintained in proliferation medium, while the remaining wells were switched to differentiation medium. Cells were fixed on day 4 for analysis. Details of medium components are listed in Table 1.

**Table 2:** Sodium current and membrane properties of OPCs in *Scn2a*^+/+^ and *Scn2a*^A263V/+^ mice

	Steady-state activation			Steady-state inactivation			Current density, pA/pF	Resting membrane potential, mV	Membrane resistance, MΩ	n
	V _1/2_, mV	k	n	V _1/2_, mV	k	n				
Scn2a^+/+^	− 20.8 ± 1.4	− 8.2 ± 0.7	18	− 62.2 ± 1.2	7.3 ± 0.3	10	− 150.5 ± 18.3	84.5 ± 3.3	146.2 ± 16.2	19
Scn2a^A263V/+^	− 20.2 ± 1.1	− 7.9 ± 0.6	20	− 56.3 ± 1.4 (*p* = 0.0045)	6.9 ± 0.3	12	− 205.4 ± 18.6 (*p* = 0.0424)	82.7 ± 3.3	151.1 ± 16.6	21

n = number of recorded cells. Data are presented as means ± SEM for normally distributed groups

### Co-culture of neurons with OPCs

Hippocampal and cortical neurons were isolated from embryonic day 17.5 (E17.5) C57BL/6N mouse embryos. Pregnant females were euthanized by CO_2_ inhalation, and embryos were immediately extracted and decapitated. Using microsurgical dissection techniques, the hippocampus or cerebral cortex was carefully isolated.

Isolated brains were washed three times with 4 °C magnesium- and calcium-free Hank’s Balanced Salt Solution (HBSS; PAA Laboratories GmbH), followed by enzymatic digestion with 2.5% trypsin for 14 min at 37 °C. The tissue was then rinsed in Dulbecco’s Modified Eagle Medium (DMEM; Biochrom AG) supplemented with fetal bovine serum (FBS; Biochrom AG), L-glutamine (Invitrogen), and penicillin/streptomycin (Invitrogen) to inactivate the trypsin.

Neurons were dissociated into single cells by gentle trituration using a pipette and filtered through a cell strainer (Becton Dickinson). For co-culturing with OPCs, 150,000 dissociated neurons were plated on 4-well Lab-Teks (Thermo Scientific). Lab-Teks were pre-coated with a poly-D-lysine (5 mg in 100 ml of HBSS, filtered through a 0.45 µm membrane) and overlaid with 500 µl of DMEM supplemented with fetal bovine serum (Biochrom AG), L-glutamine (Invitrogen), and penicillin/streptomycin (Invitrogen). After 5 h of incubation at 37 °C in a humidified 5% CO_2_ atmosphere to enable neuronal attachment, the medium was replaced with neurobasal culture medium (Invitrogen) supplemented with B27 (Invitrogen), L-glutamine, and penicillin/streptomycin. Neurons were cultured for five days before seeding OPCs (20,000 cells per well) onto them. For co-cultures, wild-type OPCs isolated from C57BL/6N mice were seeded onto neuronal cultures (10.000–11.250 cells/well). Co-cultures were initially maintained in a 1:1 mixture of enhanced neurobasal medium and OPC proliferation medium. After two days, the medium was replaced with fresh enhanced neurobasal medium and differentiation medium. On day 4, the medium was switched to neurobasal medium supplemented with myelination medium. Cultures were maintained for seven days with medium changes every second day. All cultures were kept at 37 °C in the HERAcell 150i incubator.

### Fluorescent labeling

For immunolabeling of myelin sheaths, coronal brain sections (70 µm thick) were obtained using a Leica CM1950 cryostat and were rinsed and kept in phosphate-buffered saline (PBS, Sigma-Aldrich) until used. Slices were washed twice in PBS (PBS, Sigma-Aldrich), blocked in PBS containing 1% bovine serum albumin (BSA; Gibco, 15,260) and 1% Triton X-100 (Sigma, T9284) for 1 h at room temperature. Primary antibodies were diluted in blocking solution (PBS containing 1% BSA and 0.3% Triton X-100) and applied overnight at 4 °C. The following primary antibodies were used: mouse anti-APC (1:250, Ab-7, CC1, Merck Millipore); rat anti‐myelin basic protein (MBP) (Abcam ab7349), diluted 1:250. The next day, sections were rinsed 3× in PBS, followed by incubation with secondary antibodies (donkey anti‐mouse Alexa Fluor 488, Invitrogen A21202; donkey anti‐rat Alexa Fluor 594, Invitrogen A21209; diluted 1:1000) for 1.5 h at room temperature. Nuclear counterstaining was performed using Sytox Deep Red Nucleic Acid Stain (Thermo Fisher Scientific). Slices were washed with PBS, mounted using Vectashield (Vector Laboratories), sealed with nail polish, and stored at 4 °C until imaging.

35 µm sections for cell counts in white matter were processed as follows: For antigen retrieval, slices were incubated in 10 mM citric acid (pH=6.0) at 37 °C for 1 h. After washing, slices were blocked with 0.1 M Tris-buffer saline (TBS), 3% albumin fraction V (Roth), and 0.2% Triton-X (Roth) for 1 h. Slices were incubated with the primary antibody overnight in blocking solution. The following primary antibodies were used: rabbit anti-NG2 (1:500, AB5320, Millipore), mouse anti-APC (1:250, Ab-7, CC1, Merck Millipore). After washing 3 times in TBS, secondary antibodies (goat anti-rabbit Alexa Fluor 568, 1:500, Invitrogen; goat anti-mouse DyLight 488, 1:500, Invitrogen) were applied for 3 h at room temperature. Diamidino-2-phenylindole dihydrochloride (DAPI, 0.2 mg, Sigma) was used for counterstaining of the cell nuclei, and slices were mounted in Vectashield.

OPC cultures were fixed using sequential treatment with 1% paraformaldehyde (Science Services) in PBS and, subsequently, methanol at – 20 °C, as adapted from Freeman et al. [100], followed by three 10-min washes in Dulbecco’s PBS, blocked in PBS containing 1% bovine serum albumin (BSA; Gibco, 15,260) and 0.3% Triton X-100 (Sigma, T9284) for 1 h at room temperature. Primary antibodies (rabbit anti‐Sox10, Abcam ab227680, diluted 1:200, mouse anti-Na_V_1.2 Hölzel 75–024, diluted 1:100; rat anti-MBP, Abcam ab7349, diluted 1:250; rabbit anti-NG2, Merck ZRB 5320, diluted 1:250 goat anti-PDGFRα, R&D Systems AF1062, diluted 1:250) were diluted in blocking solution (PBS containing 1% BSA and 0.1% Triton X-100) and applied overnight at 4 °C. The next day, cultures were rinsed three times in PBS and then incubated with secondary antibodies (donkey anti-mouse Alexa Fluor 488, Invitrogen A21202; donkey anti-goat Alexa Fluor 488, Invitrogen A11055; donkey anti-goat Alexa Fluor 594, Invitrogen A11058; donkey anti-rat Alexa Fluor 594, Invitrogen A21209; donkey anit-mouse Alexa Fluor 647, Jackson Immuno Research 715-605-151; donkey anti-rabbit, Alexa Fluor 647; Jackson Immuno Research 711-605-152; all diluted 1:1000) for 1.5 h at room temperature. Nuclear counterstaining was performed using DAPI (4′,6‐diamidino‐2‐phenylindole, dihydrochloride, Thermo Fisher Scientific). Labteks were kept with PBS until imaging.

For post-hoc biocytin staining, coverslips containing OPCs after patch-clamp recordings were fixed in 4% PFA for 10 min. After blocking for 1 h at room temperature (RT) with 10% normal goat serum and 0.2% Triton X-100 in PBS, the cells were incubated overnight at 4 °C with primary antibodies (rabbit anti-NG2, 1:250; mouse anti-OLIG2, 1:1000, MAB N50, Merck Millipore) and streptavidin-Cy3 (1:500, Invitrogen). The following day, cells were incubated for 1 h at RT with secondary antibodies: Alexa Fluor 488-conjugated goat anti-rabbit (1:500, Invitrogen) and Alexa Fluor 647-conjugated goat anti-mouse (1:500, Invitrogen). After washing with PBS, the coverslips were air-dried and mounted using mounting medium (SouthernBiotech).

### Fluorescence microscopy

Confocal images (50 z-stacks) of brain slices were captured using a Leica SP8 confocal microscope with a 40× oil-immersion objective (Leica Microsystems, Germany), NA 1.3, and a 0.27 µm × 0.27 µm × 0.35 µm scaling. Signals from the 488 nm laser line were detected with a filter set between 495 and 545 nm, from the 552 nm laser line between 568 and 644 nm, and from the 638-nm laser line between 691 and 788 nm, sequentially. The pinhole was set to one airy unit. For each brain slice, three images were acquired, focusing on the hippocampal CA1 region and cortical layers V-VI or I-II, depending on the age group.

Tile-scan images of the corpus callosum were obtained with a Zeiss LSM 710 confocal microscope (Zeiss, Germany) equipped with a 40x/1.3 oil-immersion objective, and a 0.415 µm × 0.415 µm × 1 µm scaling. A total of 8 stacks were collected. Signals from the 405 nm laser line were detected with a filter set between 410 and 489 nm, from the 488 nm laser line between 493 and 558 nm, and the 561 nm laser line between 564 and 639 nm.

Fluorescence imaging of cell cultures was performed with a Zeiss LSM 710 confocal microscope (Zeiss, Germany) equipped with a 40x/1.3 oil-immersion objective and a 0.415 µm × 0.415 µm × 0.5 µm scaling. Signals from the 405 nm laser line were detected with a filter set between 410 and 489 nm, from the 488 nm laser line between 493 and 558 nm, and the 561 nm laser line between 564 and 639 nm.

### Image processing and quantification

Following acquisition, myelin structures were reconstructed in 3D using Imaris 10.0 software (Oxford Instruments, UK). After conversion of lif-files, the ‘filament tracing’ tool was used to trace myelin sheaths based on the MBP (Alexa594) labeling, enabling the quantification of internode (myelin sheath) number and length assessment, as well as the mean diameter of each sheath.

Initially, a reference diameter of 0.8–1.0 µm was set using small myelin segments to standardize measurements. Once the myelin sheaths were reconstructed, a filter was applied to exclude any segments with lengths shorter than 5.00 µm and mean diameters less than 0.500 µm. This filtering process excluded the analysis of sheaths located at the edges of the analyzed volume. Following this step, the data were exported from Imaris as Excel files and later analyzed using GraphPad Prism 10.4.2 (GraphPad Software, San Diego, CA) for statistical comparisons between groups.

‘Spot tracers’ were used to identify oligodendrocyte cell bodies based on the APC (Alexa488) labeling and nuclei based on the SYTOX (Alexa638) labeling. Automated cell counts were manually verified for accuracy.

Cells of the oligodendroglial lineage are subject to tight regulation. Proliferation and differentiation of OPCs are tightly regulated and influenced by intrinsic molecular cues and extrinsic environmental signals. OPCs proliferate in response to platelet-derived growth factor (PDGF-AA) [65, 73] and can be identified by their expression of PDGFRα [99] and the surface protein NG2 [64], which are both downregulated as cells differentiate to pre-myelinating and myelinating stages [11, 35, 64]. The transcription factor Sox10, which promotes myelin gene expression and orchestrates oligodendrocyte maturation [83], is present in all oligodendroglial lineage cells [81]. Mature oligodendrocytes express markers such as myelin basic protein (MBP) and proteolipid protein (PLP) [40] or CC1 [9]. Here, we used these markers to determine the differentiating stage of oligodendroglial cells expressing either the wild-type or the gain-of-function (GOF) Na_V_1.2 variant *Scn2a*^*p.A263V*^ and to correlate expression levels with the respective state.

Na_V_1.2-channel fluorescence intensity was quantified using ImageJ software (Fiji, 2018, NIH, USA). Individual cells were manually outlined, and the mean gray value was measured for each cell following local background normalization. Three images were acquired per well, and fluorescence intensity was determined for all analyzable cells. For statistical analyses, the mean fluorescence intensity of all quantified cells per mouse was calculated, and each mouse was treated as one biological replicate. Statistical analyses were preformed using GraphPad Prism (GraphPad Software, San Diego, CA).

### Patch-clamp electrophysiology

Whole-cell patch clamp recordings on cultured OPCs were performed with an Axopatch 200 B amplifier, a Digidata 1440 A digitizer, and Clampex 10.2 data acquisition software. Currents were filtered at 10 kHz and digitized at 20 kHz. Borosilicate glass pipettes had resistances of 5–7 MΩ when filled with the pipette solution (in mM): 126 KCl, 5 EGTA, 10 HEPES, 4 Mg-ATP, 0.6 Na-GTP, 12 Na-phosphocreatine, 0.01 biocytin, pH 7.3 with KOH, osmolarity 300 mOsm/kg with mannitol. The bath solution contained (in mM): 144 NaCl, 2.5 KCl, 2 CaCl_2_, 10 HEPES, 1 NaH_2_PO_4_, 10 D-Glucose, pH 7.3 with NaOH, osmolarity 310 mOsm/kg, adjusted with mannitol. In a subset of recordings, 500 nM tetrodotoxin (TTX) was applied to confirm that the transient inward current was mediated by voltage-gated sodium channels (Supplementary Fig. 1). Quantitative analyses were performed on recordings obtained in the absence of TTX. During recordings, series resistance was compensated by 70%. Access resistance was monitored throughout the recordings, and experiments were discarded if it exceeded 10 MΩ. The residual voltage error was estimated for each recorded cell from the measured peak sodium current and the uncompensated series resistance for each recorded cell and ranged from 0.3 to 3.0 mV (mean ± SEM: 1.28 ± 0.12 mV).

Na⁺ currents were elicited by 100-ms depolarizing voltage steps from − 80 to +65 mV in 5 mV increments. Current density was calculated as peak Na⁺ current normalized to cell capacitance. The conductance–voltage relationship was derived from the current–voltage data using.

$${\mathrm{g}}\left( {\mathrm{V}} \right) = {\mathrm{I}}/\left( {{\mathrm{V}} - {\mathrm{V}}_{\mathrm{rev}}} \right).$$

Activation curves were fitted with a Boltzmann function (g(V)=g_max_/1+exp[(V-V_1/2_)/k_v_]) to obtain the half-activation voltage (V_1_/_2_) and slope factor (k_V_).

Steady-state fast inactivation was assessed using 100-ms conditioning pulses to various potentials, followed by a 5-ms test pulse to −10 mV. The peak current during the test pulse was taken as a measure of the fraction of channels available for activation. Fast inactivation curves were fitted with a Boltzmann function (I(V)=I_max_/1+exp[(V-V_1/2_)/k_v_]) to determine the half-inactivation voltage (V_1_/_2_) and slope factor (k_V_).

### In vivo conduction velocity measurement of the ventral hippocampal commissure

At P13, pups were anaesthetised with isoflurane in oxygen (4–5% for induction and 0.8–3% for maintenance) and placed in a stereotaxic frame, with the head secured using ear bars inserted into the auditory canals. Body temperature was maintained at approximately 36.5 °C using a homeothermic heating pad. Buprenorphine (0.025 mg/kg, i.p.; Temgesic, Indivior UK Limited) was administered for analgesia, and carprofen (5 mg/kg, s.c.; Norbrook Laboratories Limited, Ireland) was administered intraoperatively. The scalp was disinfected with iodine solution and removed over the surgical area. The exposed skull was cleared of connective tissue, dried with a small amount of 3% hydrogen peroxide and compressed air, and coated with OptiBond (Kerr Dental, Germany). A craniotomy was made over the cerebellum using a motorized hand drill. A steel microscrew with a pre-soldered wire was implanted into this craniotomy and served as the ground electrode. Pre-calculated implantation trajectories targeting the CA3 region [10] were marked on the skull surface for each Neuropixels probe and for the stimulation electrode. A metal headplate (Neurotar, model 5) was affixed to the skull using light-curing dental cement (Tetric EvoFlow (Ivoclar Vivadent, Germany). The ground-electrode wire was soldered to the headplate and further secured with light-curing dental cement. The remaining exposed skull was covered with two-component silicone (Kwik-Sil, WPI, USA). Following surgery, pups were removed from the stereotaxic frame and returned to their home cage with the mother and littermates.

On the following day (P14), pups were again anesthetized with isoflurane in oxygen (4–5% for induction and 0.8–3% for maintenance), positioned in a stereotaxic frame, and head-fixed via the implanted metal headplate. Silicone was removed from the skull surface, and three anteroposteriorly elongated oval craniotomies were made at the previously marked locations: two for Neuropixels 2.0 probes and one, located more laterally in the right hemisphere, for the stimulation electrode. The dura mater was incised, and craniotomies were covered with silicone oil to prevent cortical desiccation. Pups were then immediately transferred to the Mobile HomeCage setup for locomotor tracking during head-fixed movement on a lightweight, air-supported arena (Neurotar, Finland). Two Neuropixels 2.0 probes (IMEC, Belgium) and the bipolar stimulation electrode (Tungsten stereotrodes (WE3ST30.1A3, Science Products, USA), each stained with DiI and mounted on separate arms of three-axis precision manipulators (MPM; New Scale Technologies, USA), were positioned above the respective craniotomies under microscopic guidance. Neuropixels 2.0 probes were inserted at a 10° angle relative to the sagittal plane. Implantation proceeded at 50–100 μm/min under continuous electrophysiological monitoring using Open Ephys software, to a depth of 2–3 mm.

Probe advancement was halted when characteristic local field potential (LFP) and spiking activity of the CA1 pyramidal cell layer was detected approximately midway along the selected recording-channel group on both probes. The tip of each Neuropixels probe was used as the reference electrode. Once the approximate CA1 location had been identified, a new subset of recording sites distributed across two shanks was selected to enable simultaneous recordings from CA1 and CA3.

After identifying the approximate CA3 location, the stimulation electrode position was adjusted, and implantation commenced at the same rate (50–100 μm/min). To guide probe placement, a low-amplitude stimulation protocol was applied continuously during probe advancement (1 Hz, 100 μA, bipolar pulses, 100 μs pulse width, stimulator STG4002, Multi-Channel Systems, Germany). Entry into the CA3 region was inferred from the appearance of characteristic inverted sharp waves (iSPWs) in the contralateral CA1 LFP following each stimulation pulse. Upon reaching this criterion, manipulator movement was stopped. For VHC conduction velocity measurements, a train of 20 bipolar stimulation pulses was delivered to CA3 in the right hemisphere (1 Hz, 400 μA, 200 μs pulse width) to evoke repetitive high-amplitude iSPWs in contralateral CA1. At the conclusion of the experiment, pups were perfused, and brains were collected for histological analyses and validation of probe trajectories. The coronal distance between the stimulation electrode and the contralateral Neuropixels electrode was used to calculate conduction velocities from raw latencies.

### Calcium Imaging

Hippocampal slices were prepared from P4 NG2DsRed+ mice. Following rapid brain removal in cold, extracellular solution (125 mM NaCl, 4.5 mM KCl, 2 mM CaCl_2_, 1 mM MgCl_2_, 1.25 mM NaH_2_PO_4_, 26 mM NaHCO_3_, 20 mM glucose) bubbled with a mixture of 95% O_2_ and 5%CO_2_, transverse slices (550 µm) were cut and recovered for > 1 h at 34 °C. 

Slices were loaded with Cal-520™ AM (AAT Bioquest; 10 min, 37 °C) and imaged in a perfused chamber (32–34 °C) using an upright two-photon microscope (FV300, Olympus) equipped with a Ti: Sapphire mode-locked laser (Mai Tai, Spectra Physics) and a 40× water immersion objective (NA 0.8). Cal-520™ AM and DsRed were excited at 925 nm, with the emission separated at 585 nm.

Recordings were obtained from the hippocampal pyramidal layer. OPCs were identified by DsRed expression and morphology. Somatic fluorescence signals were background-corrected using adjacent regions. Regions of interest were defined in ImageJ2 (Fiji), and traces were obtained and analyzed in Igor Pro 8 (WaveMetrics).

### Statistical analysis

Extracted data were analyzed using GraphPad Prism version 10.0 (GraphPad Software, San Diego, CA, USA). Normality was assessed with the Shapiro–Wilk test. For comparisons between two groups, either the Mann–Whitney U test (for non-parametric data) or an unpaired two-tailed t-test (for normally distributed data) was used. For comparisons with more than two groups, either the Kruskal–Wallis test with Dunn’s multiple-comparison test (for nonparametric data) or ANOVA (for normally distributed data) was used. Statistical significance was defined as p < 0.05.

Conduction velocities were analyzed using a linear mixed-effects model (LMM; MATLAB, MathWorks) with genotype as a fixed effect, body weight as a fixed covariate, and animal ID as a random intercept, fitted using the *fitlme* function with restricted maximum likelihood (REML) estimation.

### Single-cell transcriptomics analysis

#### Data integration, graph construction, and oligodendroglial subtype re-annotation

We previously used single-cell transcriptomic profiles of the hippocampus from *Scn2a*^*p.A263V/*+^ mutant and wild-type mice at P4 and P14 [74]. Detailed animal protocols, generation of the engineered mouse model, and the experimental procedures are described in the original study [74]. For all analyses in this paper, we included only cells that passed quality control in the original dataset, along with their corresponding cell-type and subtype annotations.

Samples across ages and genotypes were integrated using Conos v1.5.2 [3]. Each sample was preprocessed using basicP2proc() with default parameters except min.transcripts.per.cell = 1 and n.odgenes = 2000. Inter-sample cell–cell graphs were constructed using buildGraph() with score.component.variance = TRUE, and joint dimensionality reduction was performed with UMAP using embedGraph().

To refine oligodendrocyte-lineage annotations (oligodendrocyte precursor cells (OPC), committed oligodendrocyte precursors (COP), and oligodendrocytes (OL)), oligodendrocyte-lineage cells were subsetted and clustered using the Leiden algorithm [87] via findCommunities() at resolution 1. Clusters were manually annotated based on curated marker genes and published oligodendroglial development/differentiation signatures [24, 43, 56, 72, 86, 97, 98].

#### Transcriptomic differential analysis between control and mutant conditions

Cross-condition analyses were primarily performed using Cacoa v0.4.0 [69] following the methods described in the original Cacoa publication. *Graph-based differential cell density.* Cell density was estimated on the Conos joint graph using estimateCellDensity() with method = "graph". A sample indicator matrix was smoothed with a heat kernel filter (beta = 30) and normalized by the total number of cells per sample. Differential density between genotypes was computed with estimateDiffCellDensity() using a Wilcoxon rank-sum test (type = “wilcox”), yielding signed Z-scores (positive values indicate enrichment in *Scn2a*^p.A263/+^mutants). Empirical *P* values were obtained using 400 label permutations and adjusted using the Benjamini–Hochberg correction.

*Graph-based expression shifts*. Cluster-free expression shifts were estimated on the Conos joint graph using estimateClusterFreeExpressionShifts() with gene.selection = “expression”. Expression values were smoothed over each cell’s graph neighborhood, and correlation distances were computed between samples of different genotypes. Significance was assessed with 500 genotype-label permutations, and shift magnitudes were median-filtered to match the scale of significance estimates.

*Differential expression and gene set enrichment analysis*. Pseudobulk differential expression (DE) was performed at the cell-subtype level using estimateDEPerCellType() with the DESeq2 Wald test (DESeq2 v1.38.3 [55]). Cell subtypes were required to have ≥ 10 cells per sample (min.cell.count = 10), and genes were required to be detected in ≥ 5% of cells within a cell subtype (min.cell.frac = 0.05). Independent filtering was enabled, and *P* values were adjusted using Benjamini–Hochberg correction. Gene set enrichment analysis (GSEA) was performed with estimateOntology() (type = “GSEA”) with clusterProfiler v4.6.2 [94], DOSE v3.24.2 [96], and fgsea v1.24.0 [45]. Genes were ranked by the signed DESeq2 test statistic (Z-scores), and enrichment was tested on the top 500 highly expressed genes (n.top.genes = 500). GO gene sets were obtained from org.Mm.eg.db v3.16.0, and significance was assessed using fgsea’s permutation framework with Benjamini–Hochberg correction.

#### RNA velocity-based pseudotime analysis

RNA velocity analysis was performed on oligodendrocyte-lineage cells. Spliced and unspliced UMI counts were extracted using velocyto v0.17.17 [47]. Aggregated count matrices across samples were processed with Velociraptor v1.16.0 (https://doi.org/10.18129/B9.bioc.velociraptor), interfacing with scVelo v0.2.2 [6] using the dynamical model. Library-size normalization and velocity estimation were performed with scvelo using use.theirs = TRUE. Velocity vectors were projected onto Conos-derived UMAP embeddings using embedVelocity, and grid-based velocity fields were computed with gridVectors. Pseudotime distributions between genotypes were compared using a two-sided Kolmogorov–Smirnov test and visualized using empirical cumulative distribution functions (ECDFs).

## Results

### Oligodendroglial cell composition and conduction velocity in *Scn2a*^p.A263V/+^ mice

**Fig. 1:**
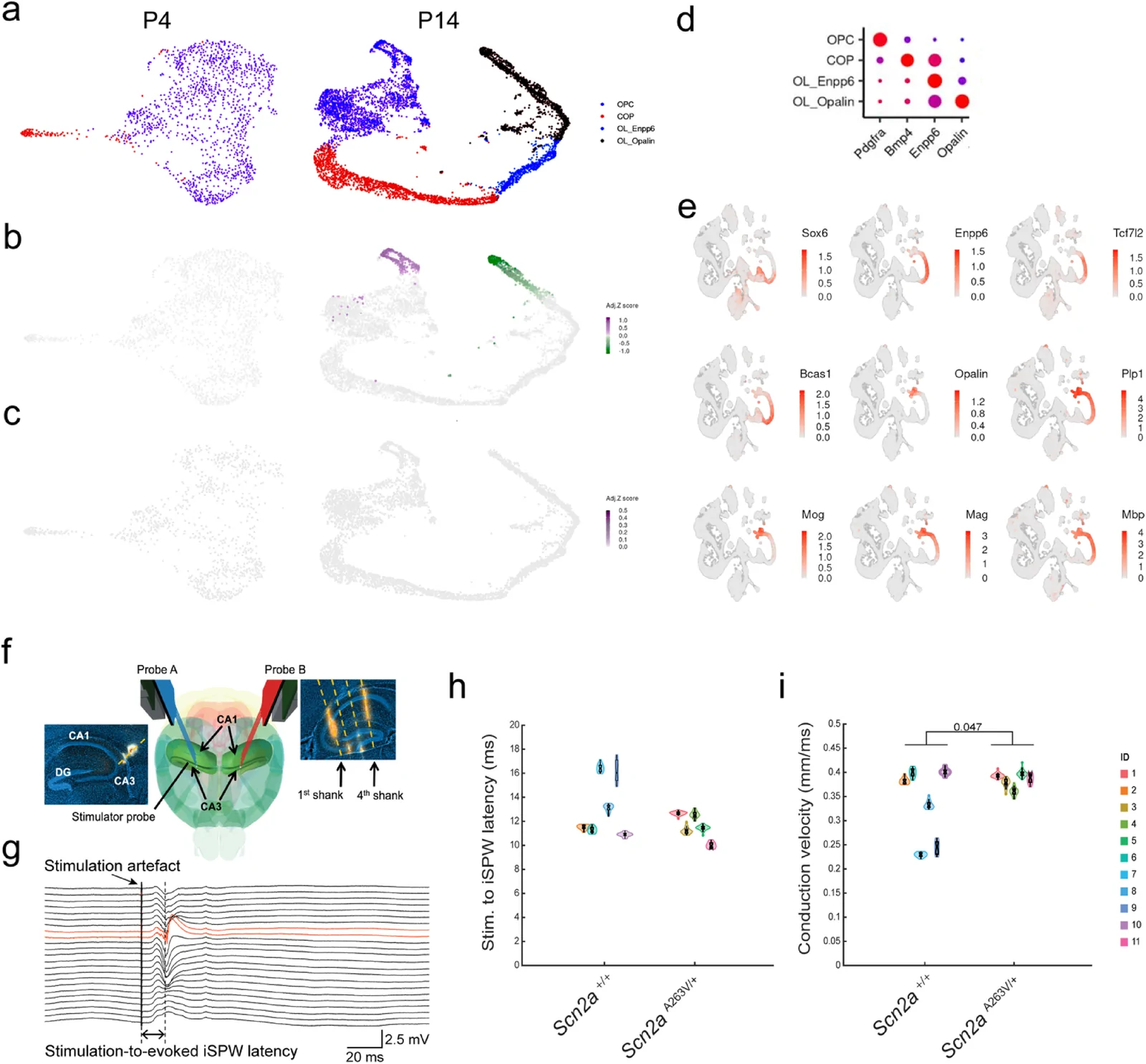
Transcriptomics comparison in oligodendrocyte-lineage subtypes and VHC conduction velocities. **a** UMAP representations of oligodendrocyte-lineage cells at P4 and P14, colored by cell subtype annotation. **b** UMAP representations of oligodendrocyte-lineage cells at P4 and P14 showing changes in cellular composition of *Scn2a*^*p.*A263V/+^ mutant cells as differences in cell density on joint graphs of oligodendrocyte-lineage cells from control and *Scn2a*^*p.*A263V/+^ mutant mice, colored by adjusted significance level. **c** UMAP representations of oligodendrocyte-lineage cells at P4 and P14 showing the extent of gene expression changes in *Scn2a*^*p.*A263V/+^ mutant cells on joint graphs of oligodendrocyte-lineage cells from control and *Scn2a*^*p.*A263V/+^ mutant mice, colored by adjusted significance level. **d** Average expression and fraction of expressing cells for selected marker genes across the oligodendroglial lineage. **e** UMAP representations of all analyzed cells, colored by expression of selected marker genes for newly formed oligodendrocytes and myelin-forming/mature oligodendrocytes; embedding was generated from the combined P4 and P14 dataset. **f** Experimental setup for measuring the VHC conduction velocity using a bipolar stimulation electrode in CA3b and bilateral Neuropixels recording electrodes oriented sagittally in the CA1-> dentate gyrus axis. DAPI-stained histological sections show DiI-stained tracks of the stimulation electrode (left) and four-shank Neuropixels 2.0 electrode (right). **g** Representative example of an evoked iSPW in dorsal CA1 (red traces indicate CA1 pyramidal layer). The latency was calculated from the time between the stimulus artifact and iSPW trough. **h** Raw latencies of 20 evoked contralateral iSPWs. Wilcoxon rank-sum test on absolute deviations from the median, MATLAB; W = 49.0, p = 0.017; n = 5 mutants, n = 6 controls. **i** VHC conduction velocities. Linear mixed-effects model, genotype fixed effect; β = −0.0249, SE = 0.0124, t(217) = −2.00, p = 0.047, 95% CI [− 0.049, −0.0004]; n = 5 mutants, n = 6 controls

To study the role of *Scn2a* in oligodendroglial cells, we used an existing single-cell transcriptomic dataset from the hippocampus of control and *Scn2a*^*p.A263V/*+^ mice, collected at two early postnatal stages (P4 and P14) [74]. We first quantified genotype-associated differences in oligodendrocyte-lineage composition and transcriptional state across development in the hippocampal formation. Joint visualization of oligodendrocyte-lineage cells across ages revealed an ordered progression from oligodendrocyte precursor cells (OPC), through committed oligodendrocyte precursors (COP) into oligodendrocyte (OL) states, with P4 dominated by OPCs and COPs and an emergence of OL populations at P14 (Fig. 1a, Supplementary Fig. 2). Within OLs, we distinguished two subtypes: (i) OL_Enpp6, characterized by high expression of *Enpp6*, *Tcf7l2*, and *Bcas1*, consistent with newly formed oligodendrocytes undergoing the transition from precursor to differentiated stages; and (ii) OL_Opalin, characterized by high expression of *Opalin*, *Plp1*, *Mog*, *Mag*, and *Mbp*, consistent with mature, myelin-forming oligodendrocytes expressing structural myelin proteins (Fig. 1d, e, Supplementary Fig. 2). This refined labeling enabled downstream analyses of genotype effects at matched developmental and cellular resolution. Graph-based differential cell-density analysis on the joint oligodendroglial embedding revealed no significant compositional changes within the oligodendroglial compartment at P4 but localized enrichment of OPCs together with depletion of OL_Opalin at P14 in *Scn2a*^*p.A263V/*+^ mice relative to controls (Fig. 1b). We did not detect significant transcriptional changes by graph-based expression shift mapping on the oligodendroglial embedding at P4 or P14 (Fig. 1c). Next, we investigated whether these subtle molecular changes affected conduction velocity in the ventral hippocampal commissure (VHC) at P14. To this end, we recorded the latency between electrical stimulation of CA3b and the trough of the evoked, contralateral inverted sharp wave (iSPW) in dorsal CA1 of awake, head-fixed mice (Fig. 1f–h). Latencies were significantly less variable in *Scn2a*^*p.A263V/*+^mice than in controls (Fig. 1h). Mean body weights, a proxy for developmental maturity, were different between the two genotype groups (p = 0.03, Mann–Whitney). Adjusting for this body weight difference, conduction velocity was slightly faster in *Scn2a*^*p.A263V/*+^ mice compared with controls (Fig. 1i). In gray matter, we did not observe differences in the density of mature (CC1-positive) oligodendrocytes in cortex and hippocampus at P15 or P60, assessed by immunohistochemical labeling of brain tissue from *Scn2a *^*p.A263V/*+^ mutant mice and controls (Supplementary Fig. 3a–d).

**Fig. 2:**
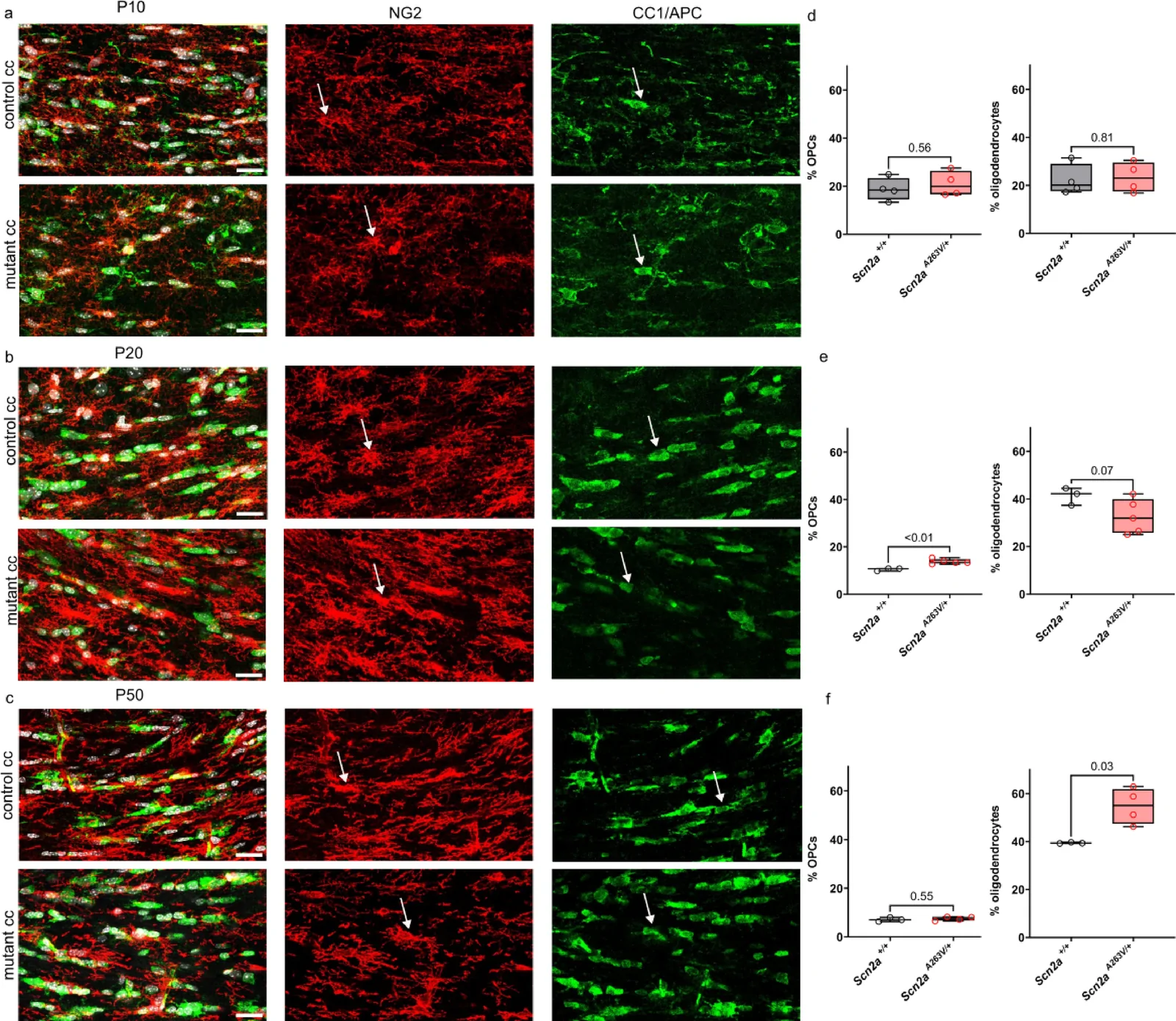
Oligodendroglial numbers in white matter in control and *Scn2a*^*p.*A263V/+^ mutant mice in different age groups **a** OPCs (labeled with anti-NG2 antibody, shown in red) and mature oligodendrocytes (labeled with anti-CC1 antibody, shown in green) in the corpus callosum of control and mutant mice at P10. **b** OPCs (labeled with anti-NG2 antibody, shown in red) and mature oligodendrocytes (labeled with anti-CC1 antibody, shown in green) in the corpus callosum of control and mutant mice at P20. **c** OPCs (labeled with anti-NG2 antibody, shown in red) and mature oligodendrocytes (labeled with anti-CC1 antibody, shown in green) in the corpus callosum of control and mutant mice at P50. **d** Assessment of the percentage of OPCs (left) and oligodendrocytes (right) across all cells in the corpus callosum at P10. About 20% of all cells are OPCs in both genotypes. Mature oligodendrocytes account for about 22% of total cells in the corpus callosum at P10 in both genotypes. N=4 per group. The difference between the 2 groups was analyzed with a Welch’s t-test (p=0.56, t=0.62, df=5.95) for OPCs and (p=0.81, t=0.24, df=6) for oligodendrocytes. **e** Assessment of the percentage of OPCs (left) and oligodendrocytes (right) across all cells in the corpus callosum at P20. About 10% of all cells are OPCs in control mice, with a significant increase to 14% in *Scn2a*^*p.*A263V/+^ mutant mice. Mature oligodendrocytes account for about 40% of total cells in the corpus callosum at P20 in both genotypes. N=3 for control mice, n=5 for *Scn2a*^*p.*A263V/+^ mutant mice. The difference between the 2 groups was analyzed with a two-tailed Welch’s test (p<0.01, t=5.65, df=5.99) for OPCs and (p=0.07, t=2.24, df=5.96) for oligodendrocytes. **f** Assessment of the percentage of OPCs (left) and oligodendrocytes (right) across all cells in the corpus callosum at P50. About 7–8% of all cells are OPCs in both genotypes. Mature oligodendrocytes represent about 40% of the total cells in the corpus callosum in control mice at P50, with a significant increase to about 55% in *Scn2a*^*p.*A263V/+^ mutant mice. N=3 for control mice, n=4 for *Scn2a*^*p.*A263V/+^ mutant mice. The difference between the 2 groups was analyzed with a Welch’s t-test (p=0.55, t=0.64, df=4.26) for OPCs and (p=0.03, t=4.10, df=3.01) for oligodendrocytes

While there was no difference in the portion of OPCs (NG2-positive) and mature oligodendrocytes (CC1-positive) among all cells in the corpus callosum at P10 (Fig. 2a,d) in the white matter, we detected a significant increase in OPCs (Fig. 2b) and a trend toward a decrease in the number of mature oligodendrocytes in *Scn2a*^*p.A263V/*+^ mice compared to controls in the corpus callosum at P20 (Fig. 2e). At P50, there were no differences in the number of OPCs, with approximately 7–8% of all cells in the corpus callosum constituting OPCs in both genotypes (Fig. 2c). However, a significant increase in mature oligodendrocytes was found in the *Scn2a*^*p.A263V/*+^ mutants as compared to controls, from 40 to 55% at P50 (Fig. 2f).

These findings suggest that there were no major changes in oligodendroglial cell numbers in the gray matter at the developmental stages assessed. However, we detected region and maturation-specific changes in the white matter of *Scn2a*^*p.A263V/*+^ mutants.

### Na_V_1.2 expression in *Scn2a*^*p.A263V/+*^ mutant OPCs

**Fig. 3:**
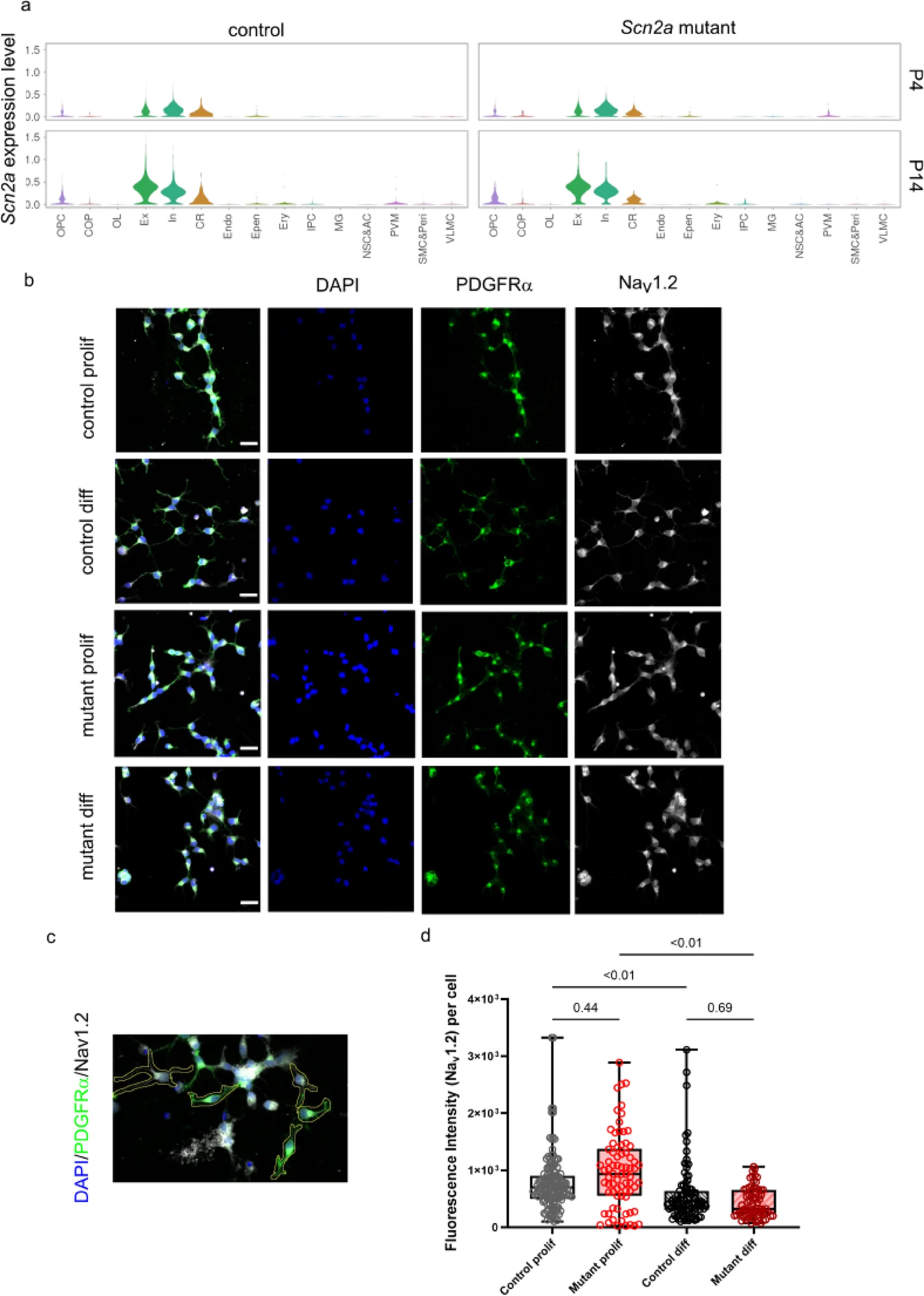
Na_v_1.2 expression in control and *Scn2a*^*p.*A263V/+^ mutant OPCs. **a** RNA expression levels of *Scn2a* across cell types in control and *Scn2a*^*p.*A263V/+^ mutant mice at P4 and P14. Abbreviations used in this figure: *OPC* Oligodendrocyte precursor cells, *COP* Committed oligodendrocyte precursors, *OL* Oligodendrocytes, *Ex* Excitatory neurons, *In* Inhibitory neurons, *CR* Cajal-Retzius cells, *Endo* Endothelial cells, *Epen* Ependymal cells, *Ery* Erythroid cells, *IPC* Intermediate progenitor cells, *MG* Microglia, *NSC&AC* Neural stem cells and astrocytes, *PVM* Perivascular macrophages, *SMC&Peri* Smooth muscle cells and pericytes, *VLMC* Vascular and leptomeningeal cells. **b** Immunocytochemical staining for Nav1.2 (shown in gray), PDGFRα (shown in green), and DAPI (shown in blue). OPCs were cultured in proliferation-promoting medium for 4 days (prolif), or for 2 days in proliferation-promoting medium and then switched to differentiation-promoting medium for 2 additional days (diff) to stop proliferation and induce differentiation using the respective growth factors (see methods for media composition). Na_V_1.2 is expressed in OPCs in proliferation-promoting medium and remains detectable during the early differentiation phase, when OPCs isolated from mutant and control mice were switched to differentiation-promoting medium. Scale bars represent 25 μm. **c** Example of the delineation of oligodendroglial cells prior to the measurement of fluorescence intensity. **d** Quantification of fluorescence intensity. 139 control OPCs in proliferation-promoting medium, 74 mutant OPCs in proliferation-promoting medium, 94 control OPCs in differentiation-promoting medium, and 71 mutant OPCs in differentiation-promoting medium were measured. OPCs were isolated from 6 control and 5 *Scn2a*^*p.*A263V/+^ mutant mice in 3 independent experiments. The Kruskal–Wallis test was performed with Dunn’s multiple comparison test (control prolif. vs. mutant prolif. p=0.44, control prolif. vs. control diff p<0.01, mutant prolif. vs. mutant diff p<0.01, control diff. vs. mutant diff. p=0.69)

To contextualize the *Scn2a*^*p.A263V*/+^ genotype in the single-cell data, we examined *Scn2a* expression across cell types and subtypes during development (Fig. 3a). As expected, *Scn2a* transcripts were detectable across major hippocampal neuronal subtypes with age- and subtype-dependent patterns. Importantly, within the oligodendrocyte lineage, *Scn2a* expression was detectable in both OPCs and COPs. It was also present at P4 and at P14. OPCs showed higher *Scn2a* expression than COPs, whereas *Scn2a* transcripts were not detectably expressed in OL_Enpp6 or OL_Opalin.

To investigate whether RNA expression translates into protein expression, we assessed Na_v_1.2 expression in OPCs maintained in stage-promoting culture media. Therefore, OPCs were purified from neonatal cortices and cultured under two different conditions: cells were either maintained in proliferation-promoting medium for four days or initially cultured in proliferation-promoting medium for two days and then switched to differentiation-promoting medium for another two days to stop proliferation and induce differentiation by adding the appropriate growth factors and signaling molecules. On day four, immunocytochemistry was performed, and the fluorescence intensity of Na_v_1.2 labeling was assessed (Fig. 3b).

To confirm the identity of purified OPCs in the cultures, we performed labeling with anti-Na_V_1.2 antibodies in PDGFRα- and NG2-positive cells (Supplementary Fig. 4). To compare the expression of Na_V_1.2, the mean fluorescence intensity derived from individual PDGFRα-positive OPCs was quantified (Fig. 3c). In OPCs isolated from control animals, Na_V_1.2 fluorescence intensity was significantly higher in those maintained in proliferation-promoting medium as compared to those that were switched to differentiation-promoting medium, potentially indicating that Na_v_1.2 is down-regulated as OPCs mature during the developmental stages (Fig. 3d). Similarly, OPCs isolated from *Scn2a*^*p.A263V*/+^ mice exhibited higher Na_V_1.2 fluorescence intensity under proliferation-promoting conditions and lower Na_V_1.2 fluorescence intensity under differentiation-promoting conditions (Fig. 3d), albeit not significant when the data were averaged per mouse (Supplementary Fig. 5).

These results support the concept that *Scn2a*-linked perturbations may be reflected not only in neurons but also in oligodendroglial precursor states.

### Developmental stage-specific expression of Na_V_1.2 channels in OPC-neuronal co-cultures

To investigate expression levels of Na_V_1.2 channels in later stages of the oligodendroglial lineage, namely pre-myelinating and myelinating oligodendrocytes, we analyzed Na_V_1.2 expression in OPCs co-cultured with neurons. While purified primary mouse OPCs do not survive well beyond four days when cultured alone, they survive and differentiate into myelinating oligodendrocytes for at least two weeks when added to a neuronal cell layer. Co-cultures with hippocampal neurons were first kept in proliferation medium for two days, then switched to differentiation-promoting medium for additional two days and subsequently maintained in myelination-promoting medium for another seven days before analysis (Supplementary Fig. 6a). In these co-cultures, oligodendroglial lineage cells were identified by the expression of the transcription factor Sox10, and pre-myelinating and myelinating oligodendrocytes by the expression of myelin basic protein (MBP). We used morphological features to further classify the developmental stages of the oligodendroglial lineage: OPCs were distinguished by the presence of two or more minimally branched processes extending from opposite sides of the cell body (Supplementary Fig. 6b). Differentiating OPCs exhibited a more complex, branched morphology but lacked MBP expression (Supplementary Fig. 6c). Pre-myelinating oligodendrocytes displayed a highly branched, tree-like structure with partial MBP expression (Supplementary Fig. 6d), while myelinating oligodendrocytes were characterized by the formation of well-defined, linear myelin sheaths as indicated by MBP immunolabeling (Supplementary Fig. 6e).

We used this classification to evaluate Na_V_1.2 channel expression in different developmental stages of oligodendroglial maturation (Supplementary Fig. 7). Na_V_1.2 was consistently detected in proliferating, differentiating, and pre-myelinating oligodendrocytes (Supplementary Fig. 7a–b). We could not detect Na_V_1.2 labeling in myelinating oligodendrocytes, and our RNA sequencing analysis consistently indicated a marked reduction in Na_V_1.2 expression with oligodendrocyte maturation.

These findings underscore a developmental-stage-specific expression pattern of Na_V_1.2, with downregulation accompanying the maturation of oligodendrocytes from pre-myelinating to fully myelinating stages. Our results confirm the temporal distribution of Na_V_1.2 channels at the RNA and protein levels, highlighting their possible functional role in the early stages of oligodendrocyte development.

### Impact of the *Scn2a*^p.A263V^ variant on OPC physiology

To assess whether expression of the GOF Na_V_1.2 variant *Scn2a*^*p.A263V*^ in OPCs alters their electrophysiological properties, we performed patch-clamp experiments to record sodium currents in control and mutant OPCs cultured in proliferation-promoting medium.

**Fig. 4:**
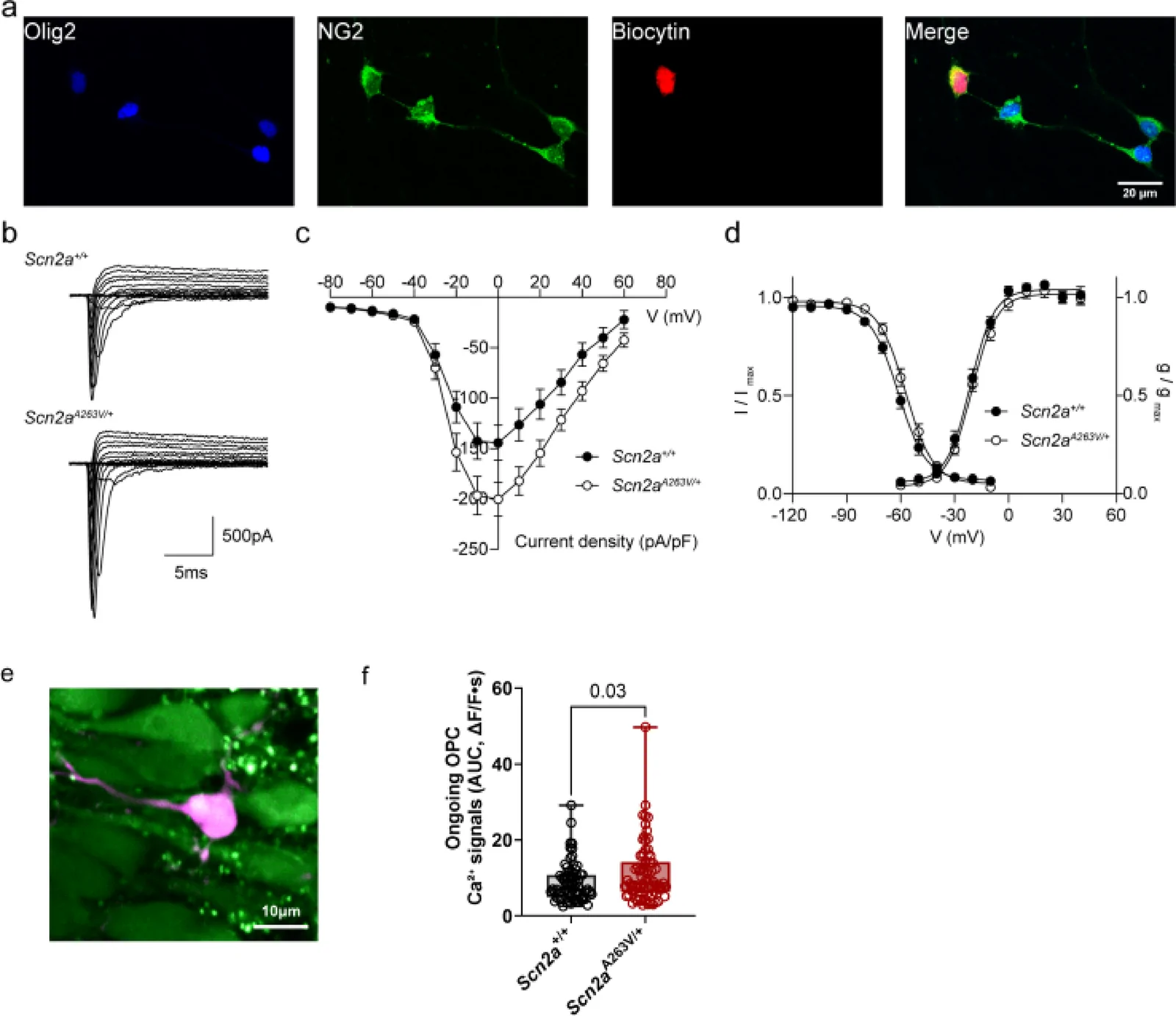
Elevated sodium currents and increased OPC calcium transients in *Scn2a*^*p.*A263V/+^ mutant OPCs as compared to control. **a** OPCs subjected to electrophysiological recording (stained with biocytin, red), indicated by co-localization with Olig2 (blue) and NG2 (green). Scale bar 20 μm. **b** Representative transient inward currents (sodium current) recorded from cultured control or mutant OPCs. **c** Peak sodium currents in OPCs (*Scn2a*^+/+^ = 19, *Scn2a*^*p.*A263V/+^ = 21) normalized to cell capacitances were plotted versus voltage. Error bars indicate SEM. Two-tailed Welch’s t-test (p = 0.04, t = 2.1, df = 37.95) was performed. **d** Voltage-dependent steady-state activation and inactivation of sodium currents recorded in OPCs. Lines represent Boltzmann functions fit to the data points. Two-tailed Welch’s t-test (p = 0.0045, t = 3.2, df = 19.92) was performed; error bars indicate SEM. **e** Representative image showing an NG2DsRed-labeled OPC (magenta) in an acute brain slice loaded with Cal-520 (green) at P4. Scale bar, 10 μm. **f** Quantification of OPC calcium signal AUC (ΔF/F·s) during a 300-s recording in wild-type and *Scn2a*^*p.*A263V/+^ mutant mice. Each dot represents one OPC, 73 OPCs from 15 control mice and 78 OPCs from 16 *Scn2a*^*p.*A263V/+^ mutant mice were analyzed. A two-tailed Mann–Whitney test was performed (p = 0.03, sum of ranks 4948, 6528)

Transient inward sodium OPCs were identified during recording by their small (~ 10 µm), round soma and multiple branched processes observed under light microscopy. Electrophysiologically, OPCs exhibited a hyperpolarized resting membrane potential (− 84.5 ± 3.3 mV), comparable to published values [12, 84], and prominent voltage-gated inward sodium currents (typically between 0.2–1 nA distinguishing them from mature oligodendrocytes, which exhibit larger outward potassium currents but lack inward sodium currents. Cell identity was subsequently confirmed by post-hoc biocytin labeling together with Olig2 and NG2 immunostaining, well-established markers of oligodendroglial cells/OPCs (Fig. 4a). Successful post-hoc biocytin labeling and NG2 immunostaining were obtained in 8 of 58 recorded cells, all of which showed co-localization with the OPC marker NG2, supporting the morphological and electrophysiological criteria used for OPC identification during recording. Transient inward sodium currents were detected in the majority of OPCs (52 in 58 recorded cells, 89.7%) (Fig. 4b). A significant increase in peak sodium current density was observed in *Scn2a *^*p.A263V/*+^ OPCs compared to *Scn2a*^+/+^ controls (Fig. 4c and Table 1), consistent with our previous findings of elevated Na_V_1.2 expression in these cells. Additionally, a depolarizing shift in the steady-state inactivation curve was observed in OPCs from *Scn2a *^*p.A263V/*+^ mice (Fig. 4d and Table 1), whereas the activation curve remained unchanged. No significant differences in resting membrane potential or membrane resistance were detected between genotypes (Table 1). Persistent sodium current was not quantified because the experimental protocol was designed to assess peak transient sodium current and voltage-dependent gating rather than persistent current.

Unlike heterologous sodium channel expression systems, cultured OPCs exhibit potassium currents that cannot be fully blocked by Cs^+^ and TEA, preventing the accurate quantification of persistent sodium currents (Fig. 4b). Overall, our data indicate that expression of the *Scn2a*^*p.A263V/*+^ variant induces a GOF effect in OPCs, consistent with results from a heterologous expression system [54].

Given the altered sodium currents observed in OPCs, we next asked whether OPC calcium signaling might be affected by changes in ion currents in *Scn2a*^*p.A263V/*+^ mice. We used NG2DsRed;*Scn2a *^*p.A263V/*+^ double transgenic mice to enable fluorescent identification of OPCs and performed calcium imaging in acute brain slices from early postnatal mice (P4) loaded with a calcium indicator (Cal-520™ AM) (Fig. 4e). Calcium signals were recorded from 151 OPCs located in the hippocampal pyramidal cell layer and surrounded by neurons, comprising 73 OPCs from control NG2DsRed;*Scn2a*^+/+^mice and 78 OPCs from NG2DsRed;*Scn2a*^*p.A263V/*+^ mice. Quantification of the calcium signal area under the curve (AUC; ΔF/F·s) during a 300-s recording revealed higher OPC calcium signal AUC in *Scn2a*^*p.A263V/*+^ mice compared with controls (Fig. 4f).

### Effect of the *Scn2a*^*p.A263V*^variant on biological processes and cellular component signatures in oligodendroglial cells

To link the expression of the *Scn2a*^*p.A263V*^ variant in the early stages of oligodendroglial cells to changes in biological mechanisms, we performed pseudobulk differential expression (DE) analysis of scRNA-seq data within each oligodendrocyte-lineage subtype. Then we applied gene set enrichment analysis (GSEA) to characterize dysregulated pathway programs in *Scn2a*^*p.A263V/*+^ mice across ages. The number of significantly enriched Gene Ontology (GO) terms among up- and downregulated genes varied by oligodendroglial states and developmental time point, predominantly in OPCs and COPs (Supplementary Fig. 8a). At P4, OPCs were characterized by predominant downregulation of protein biosynthetic and ribosome/translation programs, with metabolic programs being mildly upregulated in COPs, by contrast (Supplementary Fig. 8b).

At P14, the pattern shifted toward later-stage maturation programs. COPs showed robust upregulation of ribosome/translation and rRNA-related biosynthetic modules, while synapse/adhesion and actin/junction modules were comparatively reduced. OPCs retained prominent enrichment in mitotic and chromosome segregation processes but also showed evidence of moving away from earlier synapse/actin-associated modules. Among differentiated states, OL_Opalin also showed strong enrichment of translation/ribosome and mitochondrial programs. Notably, myelin sheath-associated enrichment was apparent at P14 (Supplementary Fig. 8c–d, red arrow).

Across P4–P14, collapsed GO clusters therefore outline a staged trajectory: early alterations emphasize suppression of OPC biosynthesis and cell-cycle programs (P4), and a later-stage program is dominated by translation/mitochondrial activity, with emergence of myelin-associated enrichment (P14). Collectively, these results support stage-specific remodeling of oligodendroglial functional states, along with a maturation-associated rebound by P14.

### Effect of the *Scn2a*^*p.A263V*^variant on myelination

**Fig. 5:**
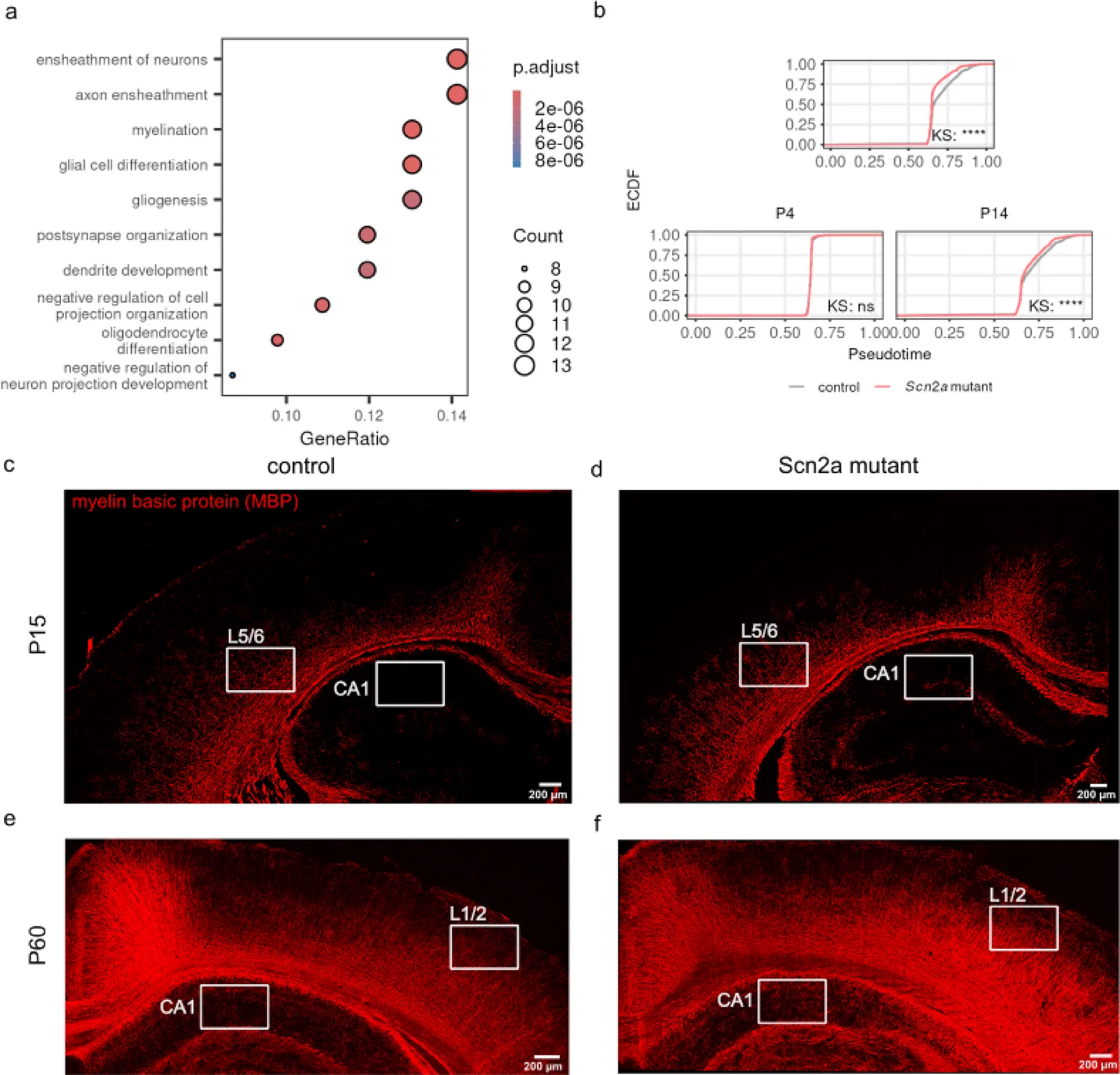
Overall changes in myelination in *Scn2a*^+/+^control and *Scn2a*^*p.*A263V/+^ mutant mice. **a** Top 10 GO BP terms enriched among the top 100 pseudotime-associated likelihood-ranked genes in oligodendrocyte-lineage cells. **b** Empirical cumulative distribution function of pseudotime for oligodendrocyte-lineage cells in control and *Scn2a*^*p.*A263V/+^ mutant mice (all ages combined). The significance of the differential distribution between genotypes was assessed using a two-sided Kolmogorov–Smirnov test. *P* values are indicated as follows: ns (*P* ≥ 0.05), * (*P* < 0.05), ** (*P* < 0.01), *** (*P* < 0.001), **** (*P* < 0.0001). Empirical cumulative distribution function of pseudotime for oligodendrocyte-lineage cells in control and *Scn2a*^*p.*A263V/+^ mutant mice stratified by age. The significance of the differential distribution between genotypes was assessed using a two-sided Kolmogorov–Smirnov test. *P* values are indicated as follows: ns (*P* ≥ 0.05), * (*P* < 0.05), ** (*P* < 0.01), *** (*P* < 0.001), **** (*P* < 0.0001). **c** Tile scan image showing overall myelination (stained with anti-MBP antibody, shown in red) in the cortex and hippocampus of control mice at P15. **d** Tile scan image showing overall myelination (stained with anti-MBP antibody, shown in red) in the cortex and hippocampus of *Scn2a*^*p.*A263V/+^ mutant mice at P15. **e** Tile scan image showing overall myelination (stained with anti-MBP antibody, shown in red) in the cortex and hippocampus of control mice at P60. **f** Tile scan image showing overall myelination (stained with anti-MBP antibody, shown in red) in the cortex and hippocampus of *Scn2a*^*p.*A263V/+^ mutant mice at P60

After detecting a stage-specific shift in maturation and myelination (Supplementary Fig. 8), we determined the Top 10 GO BP terms enriched among the top 100 pseudotime-associated likelihood-ranked genes in oligodendrocyte-lineage cells (Fig. 5a). Interestingly, the Top 3 hits were all related to ensheathment of neurons/axons and myelination. Analysis of the empirical cumulative distribution function of pseudotime for oligodendrocyte lineage cells in control and *Scn2a*^*p.A263V/*+^ mutant mice did not show differences at P4. Still, we detected a significant leftward shift at P14, indicating enrichment of earlier pseudotime states in mutants as compared to controls (Fig. 5b). Together, these data indicate stage-dependent changes in oligodendroglial progression and maturation in *Scn2a*^*p.A263V/*+^ mice by P14.

**Fig. 6:**
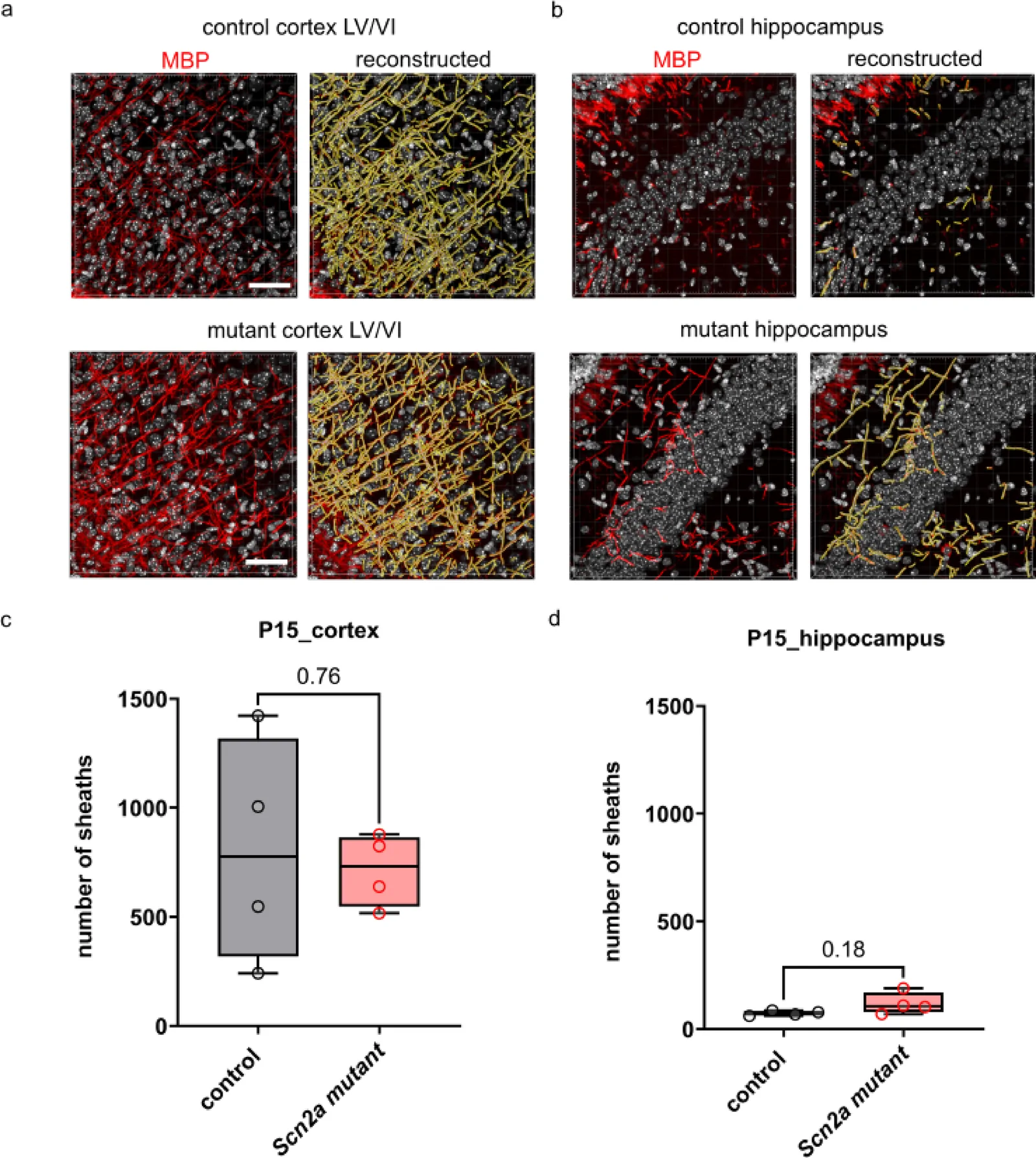
Myelin sheath numbers in the hippocampus and cortex of *Scn2a*^+/+^control and *Scn2a*^*p.*A263V/+^ mutant mice at P15. **a** The number of myelin sheaths was assessed in cortical layer V/VI at P15 in *Scn2a*^*p.*A263V/+^ mutant and *Scn2a*^+/+^control mice. The right panels represent a 3D reconstruction of myelin sheaths with Imaris. **b** The number of myelin sheaths was assessed in the hippocampal CA1 at P15 in *Scn2a*^*p.*A263V/+^ mutant and control mice. The right panels represent a 3D reconstruction of myelin sheaths with Imaris. **c** Quantification of myelin sheath numbers in layer V/VI of the somatosensory cortex at P15. Per mouse, 3 images were analyzed and averaged per mouse (n=4 for control and *Scn2a*^*p.*A263V/+^ mutant mice). Differences between mutant and control were detected with a Welch’s t-test (p=0.76, t=0.33, df=3.62). **d** Quantification of myelin sheath numbers in the CA1 region of the hippocampus at P15. Per mouse, 3 images were analyzed and averaged per mouse (n=4 for control and *Scn2a*^*p.*A263V/+^ mutant mice). Differences between mutant and control were detected with a Welch’s t-test (P=0.18, t=1.71, df=3.28). Scale bars represent 50 µm

**Fig. 7:**
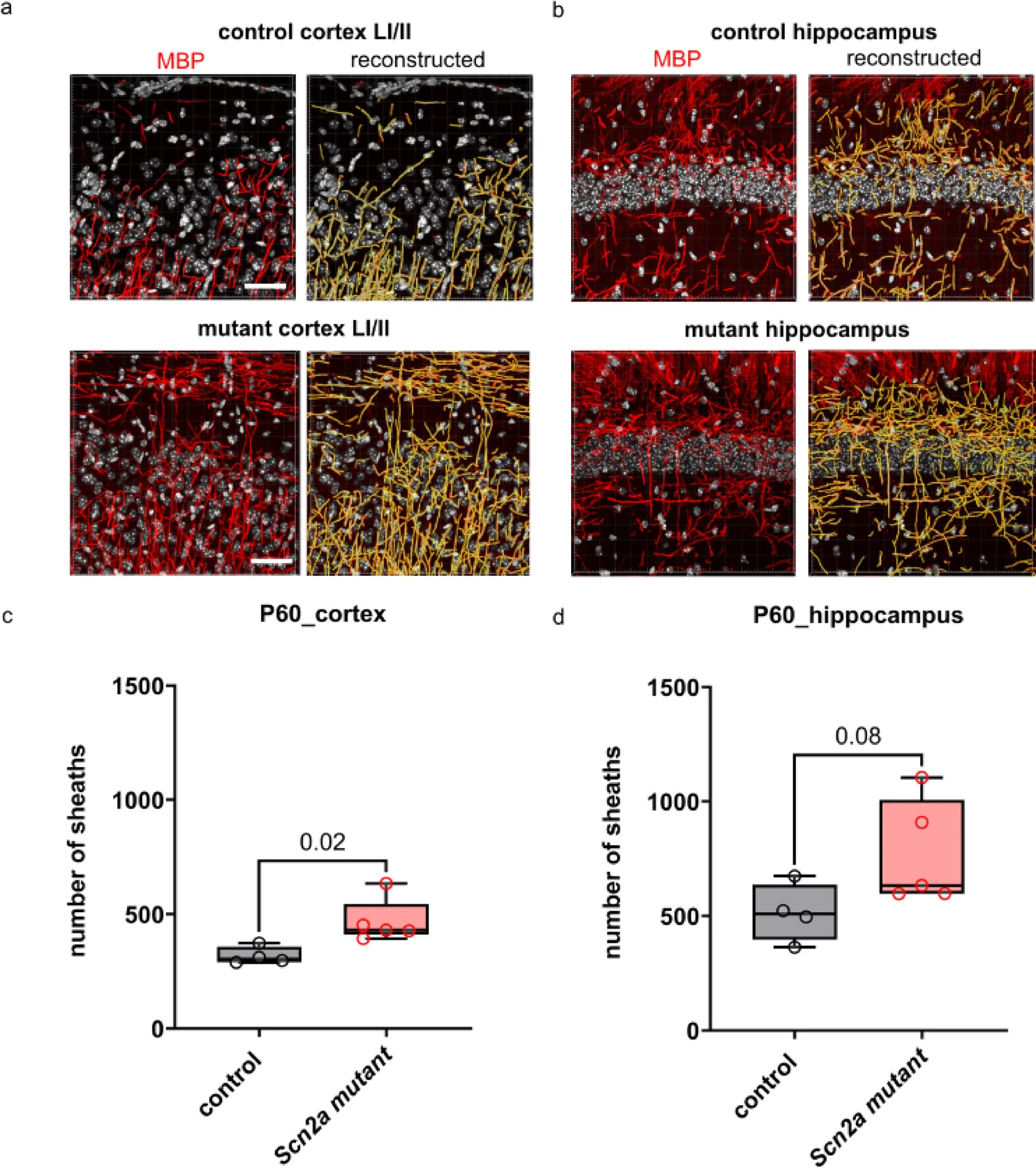
Myelin sheath numbers in the hippocampus and cortex of *Scn2a*^+/+^control and *Scn2a*^*p.*A263V/+^ mutant mice at P60. **a** The number of myelin sheaths was assessed in cortical layer I/II at P60 in *Scn2a*^*p.*A263V/+^ mutant and control mice. The right panels represent a 3D reconstruction of myelin sheaths with Imaris. **b** The number of myelin sheaths was assessed in the hippocampal CA1 at P60 in *Scn2a*^*p.*A263V/+^ mutant and control mice. The right panels show a 3D reconstruction of myelin sheaths using Imaris. **c** Quantification of myelin sheath numbers in layer I/II of the somatosensory cortex at P60. Per mouse, 3 images were analyzed and averaged per mouse (n=4 for control and n=5 for *Scn2a*^*p.*A263V/+^ mutant mice). Differences between mutant and control mice were detected with Welch’s t-test (p=0.02, t=3.21, df=5.46). **d** Quantification of myelin sheath numbers in the CA1 region of the hippocampus at P60. Per mouse, 3 images were analyzed and averaged per mouse (n=4 for control and n=5 for *Scn2a*^*p.*A263V/+^ mutant mice). Differences between mutant and control mice were detected with a Welch’s t-test (P=0.08, t=2.11, df=6.41). Scale bars represent 50 µm

We further investigated the impact of the *Scn2a*^*p.A263V*^ gain-of-function variant on myelination. Tile scan images of *Scn2a*^*p.A263V/*+^ mutant and control mice indicated an increase in overall myelination in mutant mice at P15 (Fig. 5d) and P60 (Fig. 5f) as compared to their respective controls at P15 (Fig. 5c) and P60 (Fig. 5e). To further investigate the changes in myelination, we performed a detailed analysis of myelin sheaths in gray matter regions at these two key phases of brain development: juvenile mice at P15, when myelination is ongoing, and adult mice at P60, when myelination is mostly completed. Gray matter regions were chosen for analysis because they enabled visualization and reconstruction of individual myelin sheaths, thereby allowing key myelination parameters, such as sheath diameter and segment length, to be compared between control and heterozygous mutants in both the hippocampal CA1 and cortical layers. These analyses revealed comparable internode lengths and mean diameters for all age groups and brain regions of *Scn2a*^*p.A263V/*+^ mutant and control mice, except for the hippocampal CA1 region at P15, where the length of myelin sheaths was significantly longer in mutants than in controls (Supplementary Fig. 9). When we assessed the number of myelin sheaths in the gray matter regions indicated in Fig. 5c–f, we found a trend toward higher myelin sheath numbers in *Scn2a*^*p.A263V/*+^ mutants in the CA1 region of the hippocampus at P15 (Fig. 6b, d) and P60 (Fig. 7b, d), and a significant increase in myelin sheath numbers in the somatosensory cortex at P60 (Fig. 7a, b) that was not detectable at P15 (Fig. 6a, c).

These findings indicate an increase in overall myelination attributable to the *Scn2a *^*p.A263V*^ variant of the voltage-gated sodium channel Na_V_1.2 that reaches significance at P60 and may have an impact on conduction velocity already at an early postnatal age.

## Discussion

While channelopathy research has predominantly focused on neuronal ion channel dysfunction, recent findings from patient studies and animal models indicate that myelination abnormalities are a frequent and previously underappreciated feature of the pathophysiology of neurodevelopmental diseases. Specifically, since Na_V_1.2 is expressed not only in neurons but also in oligodendroglial cells, global or OL-specific *Scn2a* haploinsufficiency in mice led to abnormal myelination and alterations in neuronal circuit function [93]. In contrast to the loss of Na_V_1.2 activity, oligodendroglial cell development and function have not been investigated in a Na_V_1.2 gain-of-function context, although white matter abnormalities were also present in patients with *SCN2A*-linked epilepsy [1].

Here, we investigated the impact of the patient-derived *Scn2a*^*p.A263V*^ gain-of-function variant on oligodendroglial characteristics in heterozygous mice and littermate controls. We analyzed expression levels of both Na_V_1.2 mRNA and protein in OPCs and report their absence in mature oligodendrocytes. We detected changes in the physiological properties of OPCs in the mutants together with changes in cell numbers in the white matter and in overall grey matter myelination in *Scn2a*^*p.A263V/*+^ mice, indicating an important role for Na_V_1.2 gain-of-function epilepsy-linked variants in the development of oligodendroglial cells and myelination.

Our analysis of a developmental scRNA-seq dataset from the hippocampus at P4 and P14 showed *Scn2a* expression in OPCs and COPs, but not in oligodendrocytes. Similarly, wild-type Na_V_1.2 protein levels were detected in OPCs maintained in proliferation-promoting medium and, to a lesser extent, in OPCs switched to differentiation-promoting medium. Yet, Na_V_1.2 expression was not observed in mature myelinating oligodendrocytes in our co-culture experiments. The difference in expression between OPCs maintained in proliferation-promoting medium and those switched to differentiation-promoting medium tended to be even more pronounced in mutants OPCs, albeit not significant, indicating a shift in OPC development in mutants, potentially towards faster maturation. At the same time, an increase in peak sodium current amplitudes and calcium transients was observed in early-stage OPCs from *Scn2a*^*p.A263V/*+^ mice. These physiological changes occurred in isolated OPCs, indicating they were independent of neuronal action potentials. Together, these findings suggest enhanced sodium channel function in mutant OPCs, potentially resulting from increased channel availability due to a depolarizing shift in steady-state inactivation. However, because native OPC sodium currents reflect the combined activity of multiple endogenous sodium channel isoforms, increased current density cannot be exclusively attributed to altered Na_V_1.2 expression or function.

Consistent with previous heterologous expression studies [54], the *SCN2A*^*p.A263V*^variant exhibited an overall GOF effect in OPCs. Specifically, we observed a depolarizing shift in steady-state fast inactivation, similar to that reported previously. In contrast, we did not detect a significant shift in activation gating. This discrepancy may reflect differences between the experimental systems used. Unlike heterologous expression systems, whole-cell sodium currents recorded from OPCs represent the combined activity of endogenous sodium channel isoforms, with Na_V_1.2 being a major but not the sole contributor to the recorded current. Furthermore, endogenous auxiliary subunits and other cell-specific regulatory mechanisms present in native OPCs may modulate channel gating, resulting in subtle differences compared with recombinant expression systems. Additional factors, including cell-specific differences in channel trafficking, membrane localization, and regulatory interactions, may further influence Na_V_-channel properties in native OPCs and contribute to differences observed across experimental systems.

A limitation of the present study is that persistent sodium current was not quantified. Reliable assessment of persistent sodium current requires systematic pharmacological isolation, for example by TTX subtraction or voltage-ramp protocols performed before and after TTX application. Moreover, in native OPCs, persistent sodium currents would represent the combined contribution of multiple endogenous sodium channel isoforms rather than Na_V_1.2 alone, limiting the ability to directly attribute this current component to the *Scn2a*^*p.A263V*^variant. This limitation is particularly relevant because peak sodium currents in native OPCs are substantially smaller than those typically observed in heterologous expression systems, making the expected persistent current comparable in magnitude to residual leak and incompletely blocked non-sodium currents. Therefore, although our findings demonstrate altered transient sodium current properties consistent with a GOF effect, additional experiments specifically designed to isolate persistent sodium currents will be required to determine whether this current component is altered in native OPCs.

Overall, our findings extend earlier physiological studies by demonstrating the presence of sodium currents and mRNAs encoding voltage-gated sodium channels in OPCs and premyelinating oligodendrocytes, but not in mature oligodendrocytes [18]. Given that Na_V_1.2 is expressed only in the early stages of oligodendroglial development, cell-autonomous effects of pathogenic variants are likely to occur only in OPCs and COPs, but not in mature oligodendrocytes. As OPCs mature in the mouse brain, Na_V_-channel density increases sharply after birth, peaking at about P12 and plateauing by P80 [82]. Accordingly, the strongest effect of Na_V_1.2 variants is expected to occur during early postnatal brain development.

Calcium signals in OPCs have been shown to regulate proliferation and differentiation of OPCs [15, 52, 79], which is highly dependent on the respective context. Analysis of the scRNA-seq dataset and immunohistochemical labeling of brain tissue from *Scn2a*^*p.A263V/*+^ mice and controls did not reveal significant changes in the cellular composition or the developmental stage distribution of oligodendroglial cells. Thus, the changes in physiological properties caused by the Na_V_1.2 gain-of-function did not affect proliferation or differentiation. Although we detected an increase in the biological process (BP) terms of *glial cell differentiation* and *gliogenesis* in pseudotime analysis of the hippocampus, there were no major changes in oligodendrocyte numbers in gray matter regions of mutant mice in the age groups analyzed. By contrast, in the white matter of *Scn2a*^*p.A263V/*+^ mice, we found an increase in OPCs at P20 and an increase in mature oligodendrocytes at P50. These findings indicate that the impact of this variant on oligodendroglial cell numbers is region dependent. These regional differences are caused by the microenvironments OPCs encounter, which generally lead to higher OPC proliferation and differentiation rates in white matter than in gray matter [80].

Synaptic input and concomitantly evoked calcium events at microdomains on OPC processes have recently been shown to predetermine myelination patterns in zebrafish [53].

Thus, it is possible that the elevated sodium current and calcium transients observed in cells from *Scn2a*^*p.A263V/*+^ mice promoted OPC differentiation and subsequent myelination, or, alternatively, that the increased frequency of localized spontaneous Ca^2^⁺ transients within nascent process microdomains of mutant OPCs acted through actin-dependent cytoskeletal remodeling to accelerate myelin sheath extension and consolidation. The mechanistic details of calcium signaling changes in mutant OPCs during development and their relationship to increased myelination remain to be studied.

Pseudotime-associated likelihood-ranked genes in oligodendrocyte-lineage cells revealed an upregulation of myelination and ensheathment of neurons/axons among the top ten GO BP terms in the mutants. Additionally, we detected a leftward shift in the pseudotime analysis, indicating accelerated development, a pattern also observed in hippocampal neurons [74]. While early OPC dysregulation does not necessarily reduce myelination, it can reshape the timing and extent of myelination. This finding is consistent with earlier studies showing that elevations in intracellular ion signaling in cultured OPCs influenced MBP synthesis [30]. Accordingly, our myelination analysis in cortex and hippocampus showed stronger myelination in *Scn2a*^*p.A263V*/+^ mice, with significantly more myelin sheaths in the upper layers of the somatosensory cortex. Taken together, our results indicate that the increase in myelination is caused by an increased capacity to myelinate by each oligodendrocyte, rather than by an increase in their numbers.

Myelination of the cortex begins in the deeper cortical layers before it reaches the upper cortical layers in adult mice [26, 68, 89]. Since layer 1/2 did not contain any myelin sheaths at P15, layer 5/6 was analyzed, but no significant differences were detected. At P60, we compared myelin sheath numbers in layer 1/2 between control and mutant mice. At that stage, *Scn2a*^*p.A263V*/+^ mutant mice had significantly more myelin sheaths than controls. Overall, we did not find any differences in the mean myelin sheath diameter across all samples analyzed with our method. Still, there was a significant increase in myelin sheath length in the mutant hippocampus at P15. These differences in myelination observed at P15 and P60 could be linked to a combination of developmental and activity-dependent mechanisms. Yet, both neurons and OPCs predominantly express the Na_V_1.2 isoform in early development. Although we did not detect Na_V_1.2 (RNA and protein) expression in mature, myelinating oligodendrocytes, Na_V_1.2 is expressed in OPCs and COPs during early development, and the *Scn2a*^*p.A263V/*+^ variant altered Na_V_1.2 expression dynamics and OPC physiology. Thus, early cell-autonomous disturbances in oligodendroglial maturation may have persistent consequences for later myelin formation. In parallel, chronic alterations in neuronal network activity may drive secondary adaptive remodeling of glial cells and myelination. A recent study in Dravet syndrome further illustrates that genetic epilepsy can be accompanied by long-lasting remodeling of glial cells [32]. Together, our findings indicate that the increased myelination in *Scn2a*^*p.*A263V/+^ mice may arise from a combination of early developmental alterations within the oligodendroglial lineage. At a later stage, the changes in myelination are a likely compensatory mechanism in response to the disturbances in the underlying neuronal network [37, 74, 78]. In support of this hypothesis, it is well established that neuronal activity promotes myelin formation [33, 38, 57, 58, 66, 90]. We did not observe an increase in oligodendrocyte numbers in the cortex or hippocampus, indicating that the increase in myelin sheaths is attributable to an increase in the number of internodes generated per oligodendrocyte. By contrast, the number of oligodendrocytes in the white matter of adult mice was increased, likely contributing to changes in the myelin compartment.

Myelination enables saltatory conduction (reviewed in [42]), metabolically supports axons [31, 60, 62], and is plastic throughout life [19], with the myelin content increasing throughout childhood and adolescence [49, 50]. Altered myelin features will change the properties and conduction velocities of the underlying neurons, as demonstrated in the auditory system [27]. Adaptive remodeling of myelination profiles in response to sensory input in the neocortex was shown to diversify circuit tuning [95]. The changes in myelination patterns we observed in *Scn2a*^*p.A263V/*+^ mice could alter the timing and efficiency of action potential propagation, thus influencing conduction velocities, synaptic plasticity, functional connectivity, and neuronal synchronization. In line with earlier reports on myelination properties having an influence on the timing of action potential propagation, we observed less variable and slightly increased conduction velocities of the VHC in the hippocampus of infantile mice, supporting the functional significance of hypermyelination in mutants. It is further tempting to speculate that the altered myelin architecture contributes to the beta/low-gamma frequency synchronization at seizure onset in the hippocampus of *Scn2a*^*p.A263V/*+^ mice.

Maladaptive myelination was described in another mouse model of epilepsy in which a *Scn8a* loss-of-function was introduced. In this model, increased myelin thickness and oligodendrogenesis contributed to epilepsy progression, which was prevented by pharmacological inhibition of oligodendrogenesis [44]. Myelination was also compromised in focal cortical dysplasia type IIa [21] and after kainic acid-induced adolescent seizures [28]. Given the neural network’s plasticity, myelination may be differentially affected by channel type, developmental time point of expression, and the specific mutation leading to epilepsy, exerting a specific influence on the neuronal network.

OPCs express sodium channels, including Na_V_1.2 [41, 82, 88]. Electrical properties such as potassium conductance influence OPC behavior and determine their proliferative potential [16, 71]. The downregulation of voltage-gated sodium channels is also linked to the loss of synaptic input from neurons onto oligodendroglial cells [46]. Subtle changes in electrical properties likely alter communication between neurons and oligodendroglial cells. Previous work by Kim and colleagues identified a subset of immature premyelinating oligodendrocytes with elevated Na_V_1.2 expression. It demonstrated that deletion of Na_V_1.2 in these cells significantly reduced OL-axon interactions and impaired myelination [8]. Importantly, these excitable premyelinating OLs not only existed early in development but persisted into adulthood. Deletion of *Scn2a*, specifically in oligodendroglial cells, impaired their maturation [34]. Together, these data reinforce the emerging view that Na_V_1.2 channels are not only present but functionally important during early OPC development and the progression toward myelination. Moreover, the conditional loss of *Scn2a* in oligodendroglial cells impairs myelination without affecting axon caliber in the auditory system. Still, it alters sodium channel expression in axons and the intrinsic excitability at nerve terminals, leading to conduction failures and impairing auditory function in an ASD model [2]. It is possible that a gain-of-function variant of *Scn2a* has the opposite effect, supporting oligodendroglial maturation. Although we did not observe major changes in cellular composition in the hippocampal scRNA-seq dataset, the increase in mature oligodendrocytes in adult mice and in OPCs at P20 in the white matter as well as an increase in average sheath length at P15 in the hippocampus of *Scn2a*^*p.A263V/*+^ mice may contribute to pathological network function in this model. While the increase in myelination of the somatosensory cortex in adult mice may reflect the network hyperexcitability observed in this model, our data clearly support a direct cell-autonomous effect of the *Scn2a* gain-of-function variant on OPC physiology.

## Supplementary Information

Below is the link to the electronic supplementary material.

Supplementary Material 1

## Data Availability

Data are available upon reasonable request.
