# Supplementary material for: Altered oligodendroglial cell development and increased myelination in SCN2A gain-of-function-linked epilepsy
Source: Acta Neuropathol Commun. 2026 Oct 7;14:212. doi: 10.1186/s40478-026-02437-1 (PMC13651182; doi:10.1186/s40478-026-02437-1)
Supplement: Supplementary file 1 [file 40478_2026_2437_MOESM1_ESM.pdf]

Supplementary Figures:

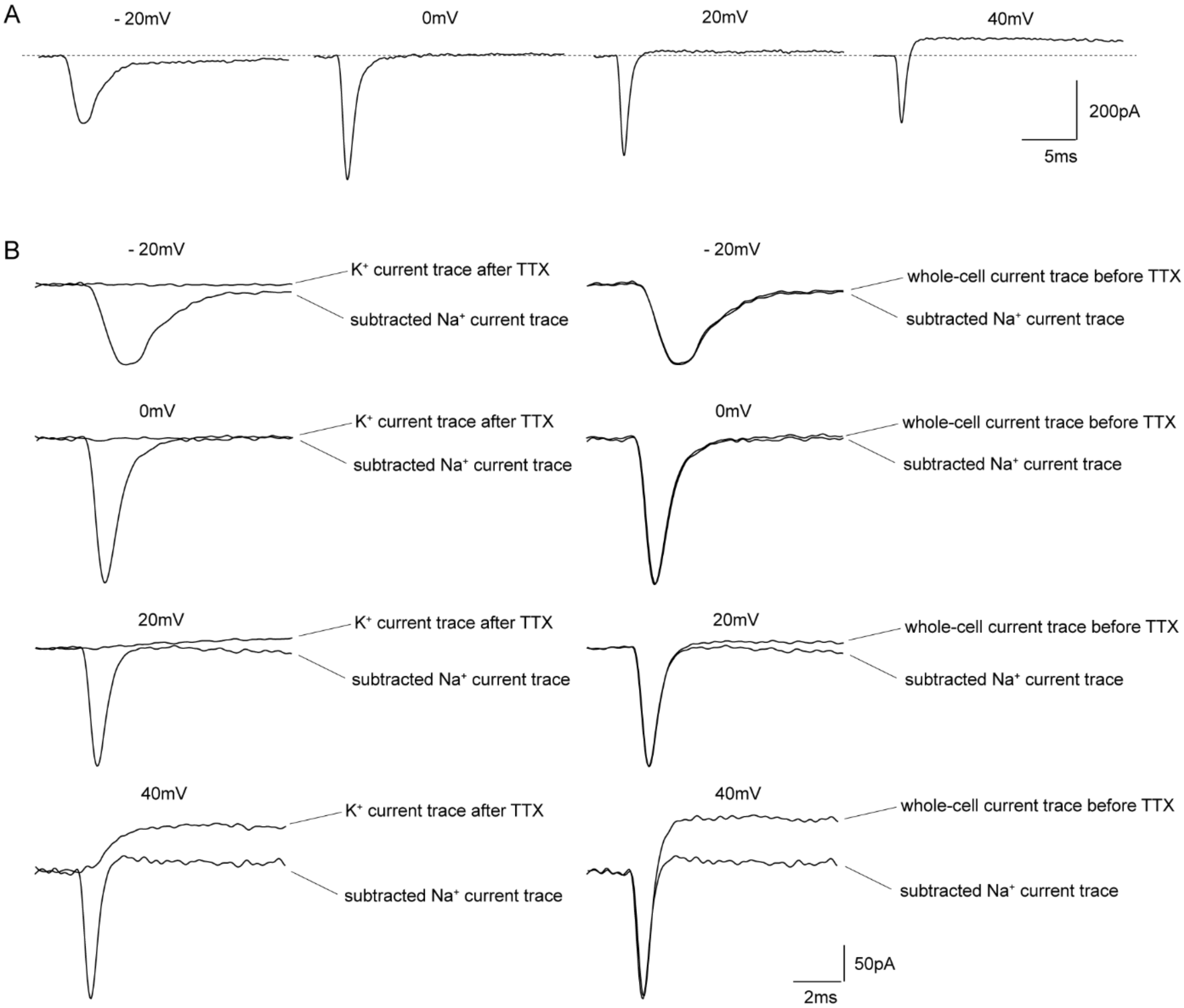

Supplementary Figure 1: Residual potassium currents minimally influence measurements of peak transient sodium current.

a: Representative whole-cell current traces recorded from an OPC at different depolarizing potentials. Residual outward potassium currents remained minimal over the voltage range at which peak transient sodium current was measured (−20 to +40 mV) and became more prominent only at more depolarized potentials.

b: Representative recording obtained at different depolarizing potentials, illustrating the different scenario for contamination by residual potassium currents. Left, comparison of the current trace recorded after TTX application (residual potassium current) with the TTX-subtracted sodium current. Right, comparison of the original whole cell current trace recorded before TTX application with the TTX-subtracted sodium current. Even under +40mV, the residual potassium current had only a

minimal effect on the amplitude and kinetics of the peak transient inward sodium current, indicating that measurements of peak sodium current and voltage-dependent gating are only minimally affected by residual potassium conductance under the present recording conditions.

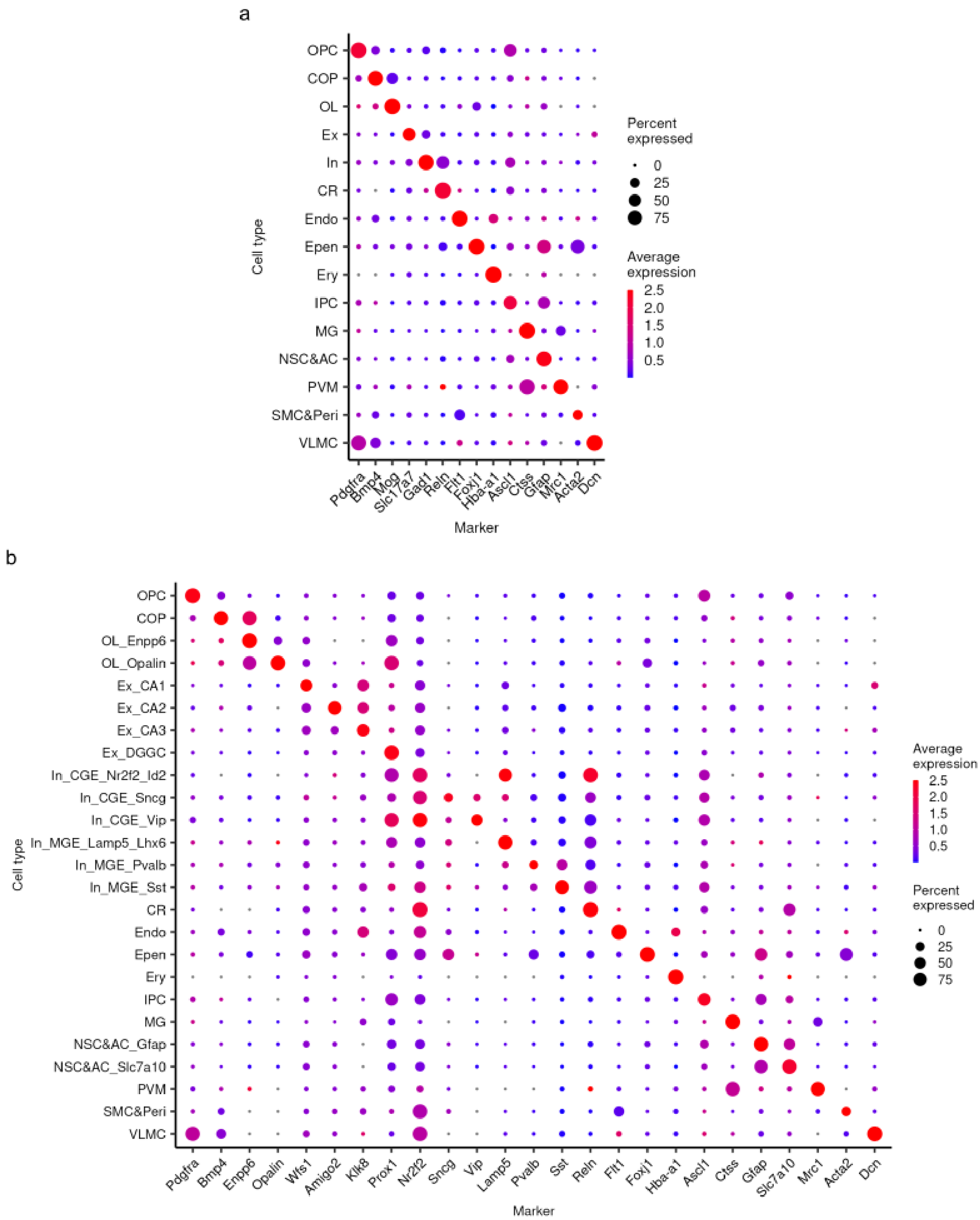

Supplementary Figure 2: Marker gene profiles across cell types and subtypes

a: Average expression and fraction of expressing cells for selected marker genes across cell types.

b: Average expression and fraction of expressing cells for selected marker genes across cell subtypes.

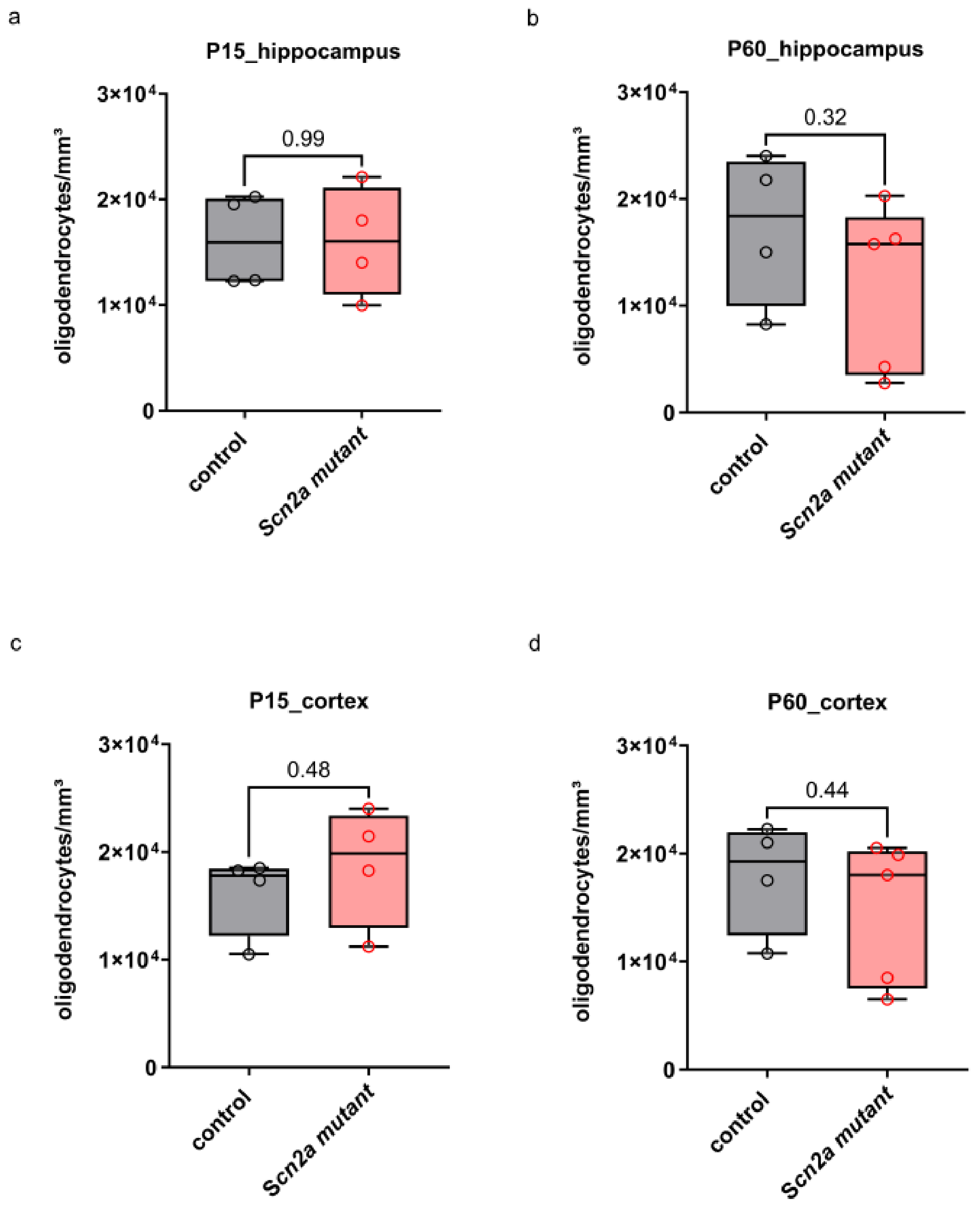

Supplementary Figure 3: **No changes in oligodendrocyte density in the gray matter in *Scn2a*$^{+/+}$ control and *Scn2a*$^{p.A263V/+}$ mutant mice in different age groups**

a: CC1-positive OLs in the hippocampal CA1 at P15 in control and heterozygous *Scn2a*$^{p.A263V/+}$ mutant mice. N=4 mice per group. Normality was assessed with Shapiro-Wilk test, and the difference between the 2 groups was analyzed with two-tailed Welch's test (p=0.99, t=0.02, df=5.83). There is no difference in cell numbers between genotypes.

b: CC1-positive OLs in the hippocampal CA1 at P60 in control and heterozygous *Scn2a*$^{p.A263V/+}$ mutant mice. N=4 for control and n=5 for *Scn2a*$^{p.A263V/+}$ mutant mice. Normality was assessed with Shapiro-

Wilk test, and the difference between the 2 groups was analyzed with two-tailed Welch's test (p=0.32, t=1.08, df=6.83). There is no difference in cell numbers between genotypes.

c: CC1-positive OLs in layer V/VI of the somatosensory cortex at P15 in control and heterozygous *Scn2a*$^{p.\text{A263V/+}}$ mutant mice. N=4 mice per group. Normality was assessed with Shapiro-Wilk test, and the difference between the 2 groups was analyzed with two-tailed Welch's test (p=0.48, t=0.77, df=5.31). There is no difference in cell numbers between genotypes.

d: CC1-positive OLs in layer I/II of the somatosensory cortex at P60 in control and heterozygous *Scn2a*$^{p.\text{A263V/+}}$ mutant mice. N=4 for control and n=5 for *Scn2a*$^{p.\text{A263V/+}}$ mutant mice. Normality was assessed with Shapiro-Wilk test, and the difference between the 2 groups was analyzed with two-tailed Welch's test (t=0.44, t=0.81, df=7). There is no difference in cell numbers between genotypes.

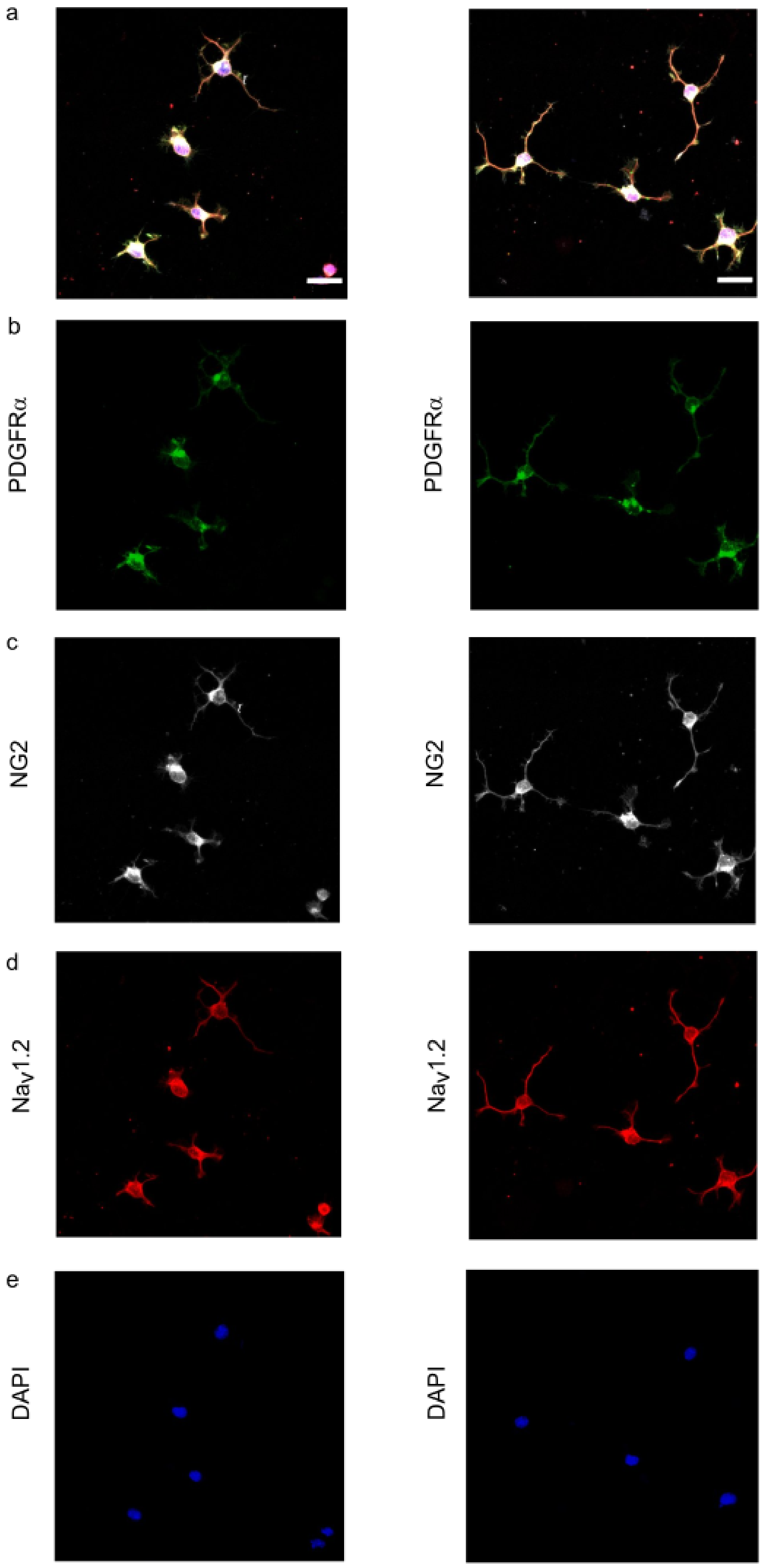

Supplementary Figure 4: Identification of purified, primary, cortical OPCs

a: examples of OPCs purified from neonate cortices by the immune-panning procedure

b: labeling with anti-PDGFRα antibody (shown in green)

c: labeling with anti-NG2 antibody (shown in gray)

d: labeling with anti-$Na_V1.2$ antibody (shown in red)

e: DAPI is shown in blue

Scale bars represent 25µm.

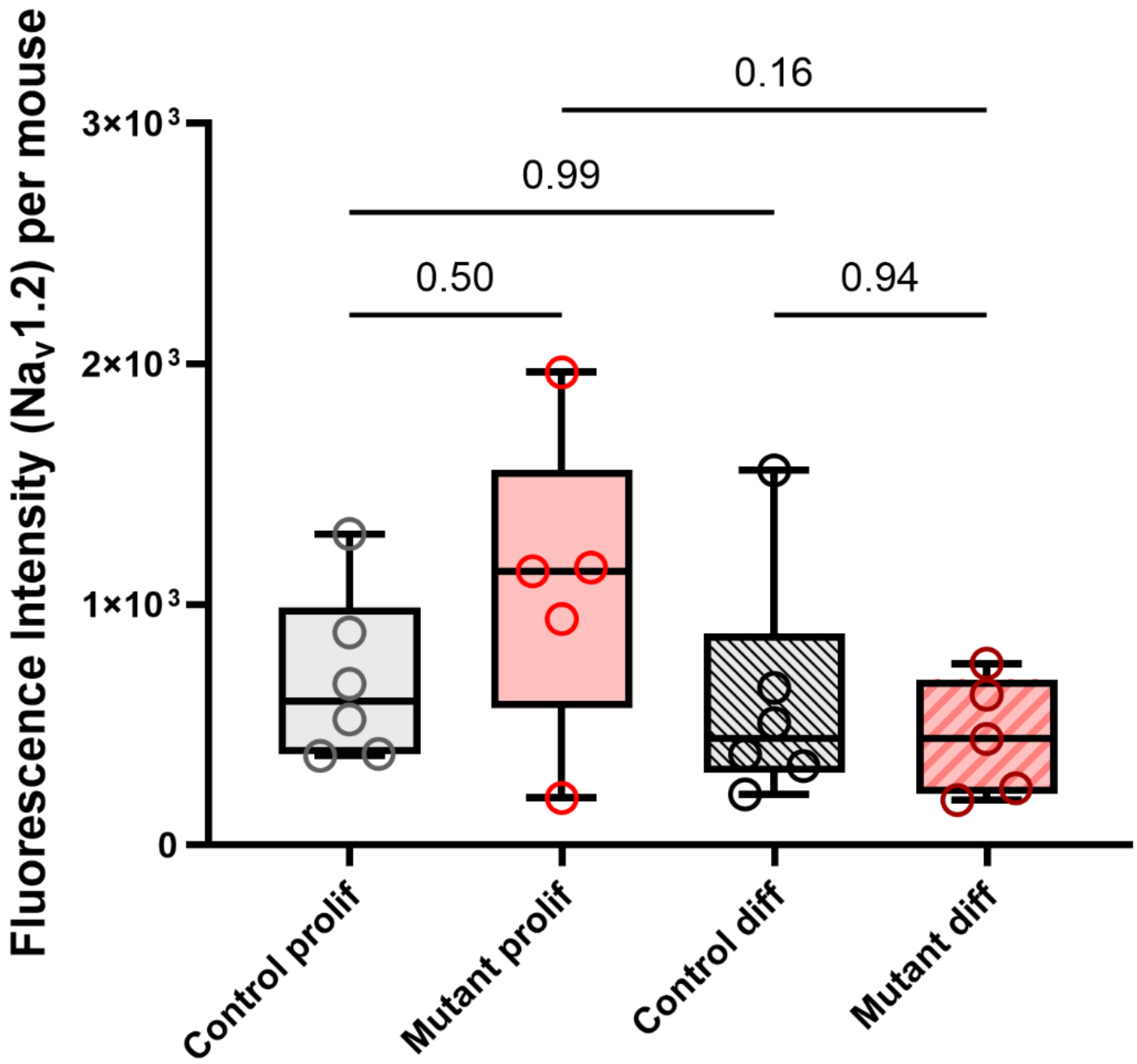

Supplementary Figure 5: Na_V1.2 expression in OPCs averaged per mouse

Individual data points represent the mean luminescence value per mouse across Scn2a+/+control (n=6) and Scn2ap.A263V/+mutant (n=5) mice. Data are presented as a box plot layout displaying the frequency distribution, median (center line), interquartile range (box limits), and total range from minimum to maximum values (whiskers). Statistical analysis was performed using a one-way ANOVA ($F (3, 18) = 1.780$) with Tukey´s multiple comparison test. No statistically significant differences in fluorescence intensity were observed between control and mutant groups overall ($p=0.19$), comparison between experimental groups are displayed in the graph.

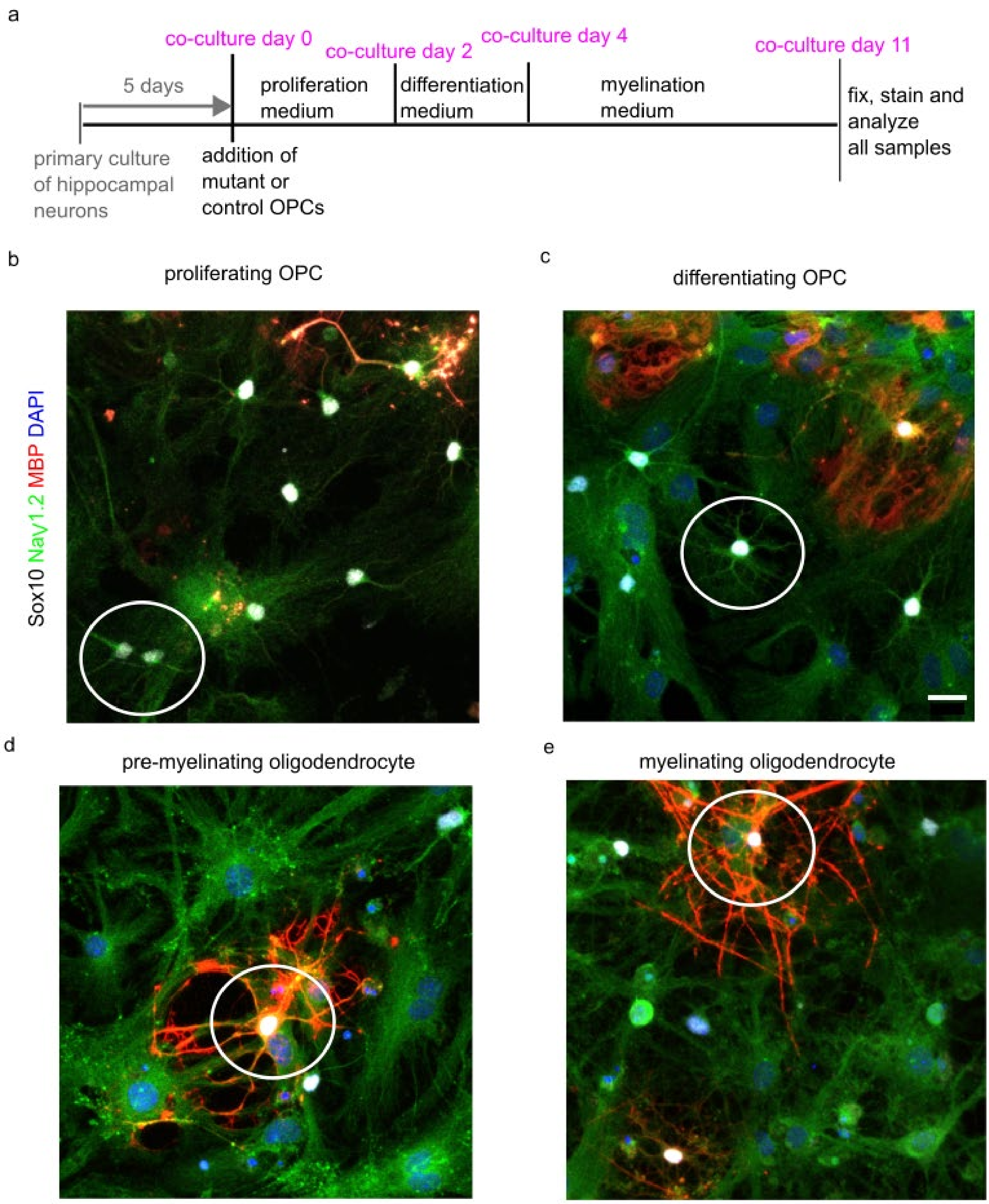

Supplementary Figure 6: Classification of oligodendroglial cells in co-cultures.

a: experimental timeline. OPCs were cultured on hippocampal neurons in proliferation medium for 2 days, then switched to differentiation medium for another 2 days, and then kept in myelination medium for an additional 7 days to allow for myelination.

b: encircled cells were classified as proliferating cells, characterized by two processes extending from the cell body.

c: encircled cell was classified as differentiating cell, identified by its branched, tree-like structure without MBP expression.

d: encircled cell was classified as pre-myelinating oligodendrocyte, distinguished by its branched morphology and the expression of MBP.

e: encircled cell was classified as myelinating oligodendrocyte with well-formed, clearly identifiable myelin sheaths.

All oligodendroglial cells are identified by Sox-10 labeling (shown in grey), Na_V1.2 is shown in green, MBP is shown in red.

Scale bar represents 20 µm.

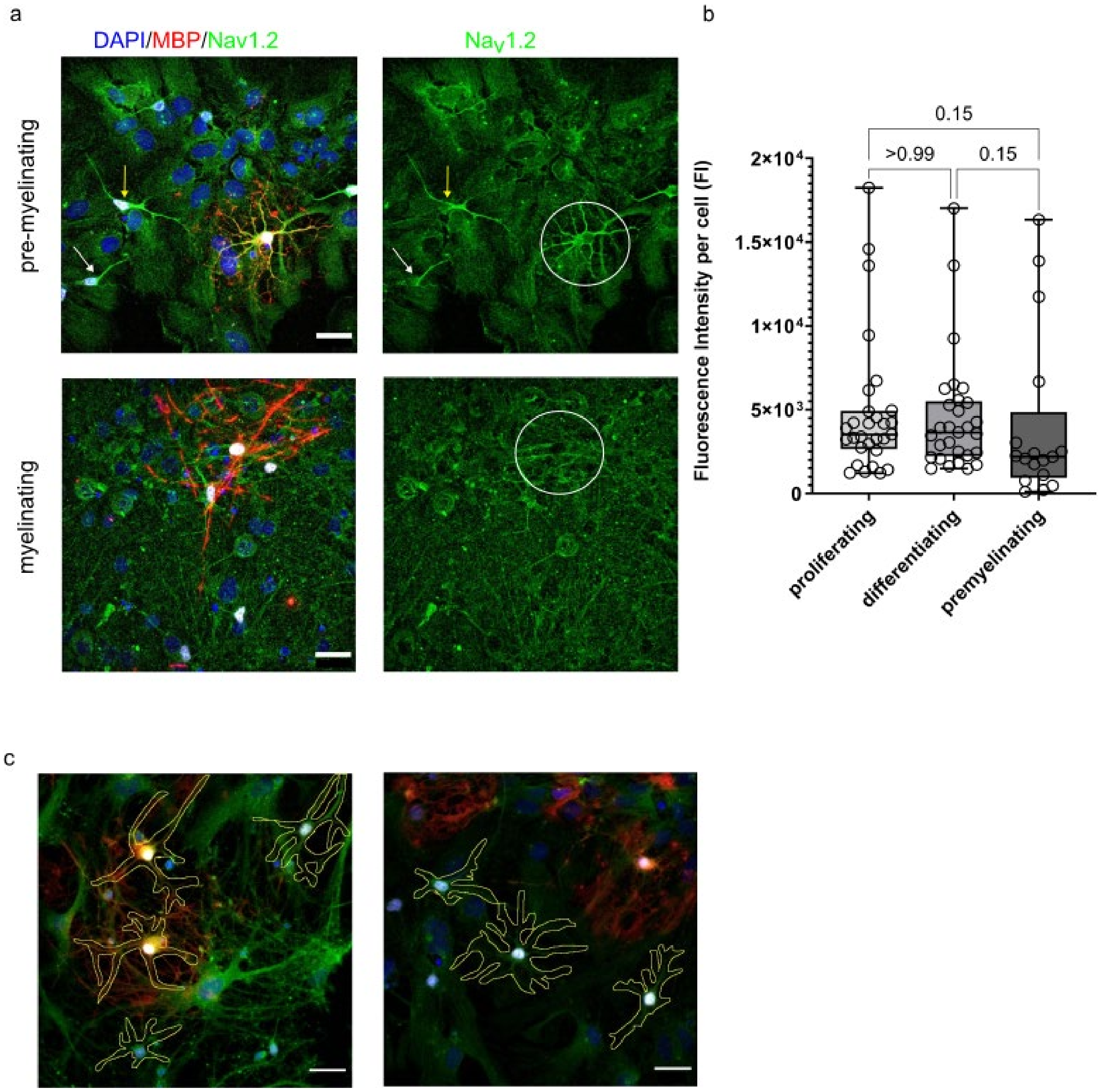

Supplementary Figure 7: **Developmental stage-specific downregulation of Na_V1.2 channels during oligodendrocyte maturation**

a: Na_V1.2 fluorescence intensity occurs in proliferating (white arrow) and differentiating OPCs (yellow arrow) and as oligodendrocytes mature, in pre-myelinating oligodendrocytes (white circle, upper right image). Na_V1.2 fluorescence intensity is not detectable in myelinating oligodendrocytes (white circle lower right image).

Scale bars represent 25 µm.

b: Quantification of Na_V1.2 channel expression in co-cultures. Fluorescence intensity per cell was analyzed. Data demonstrates comparable expression of Na_V1.2 in proliferating and differentiating OPCs as well as in pre-myelination oligodendrocytes. Individual values were plotted as a means with standard deviation (SD) and error bars shown. 29 proliferating OPCs, 29 differentiating OPCs and 17

pre-myelinating oligodendrocytes were measured. OPCs were isolated from 9 C57BL/6 mice in 3 independent experiments. Kruskal-Wallis test was performed with Dunn's multiple comparison test.

c: Examples for the delineation of oligodendroglial cells prior to the measurement of fluorescence intensity. Scale bars represent 25µm.

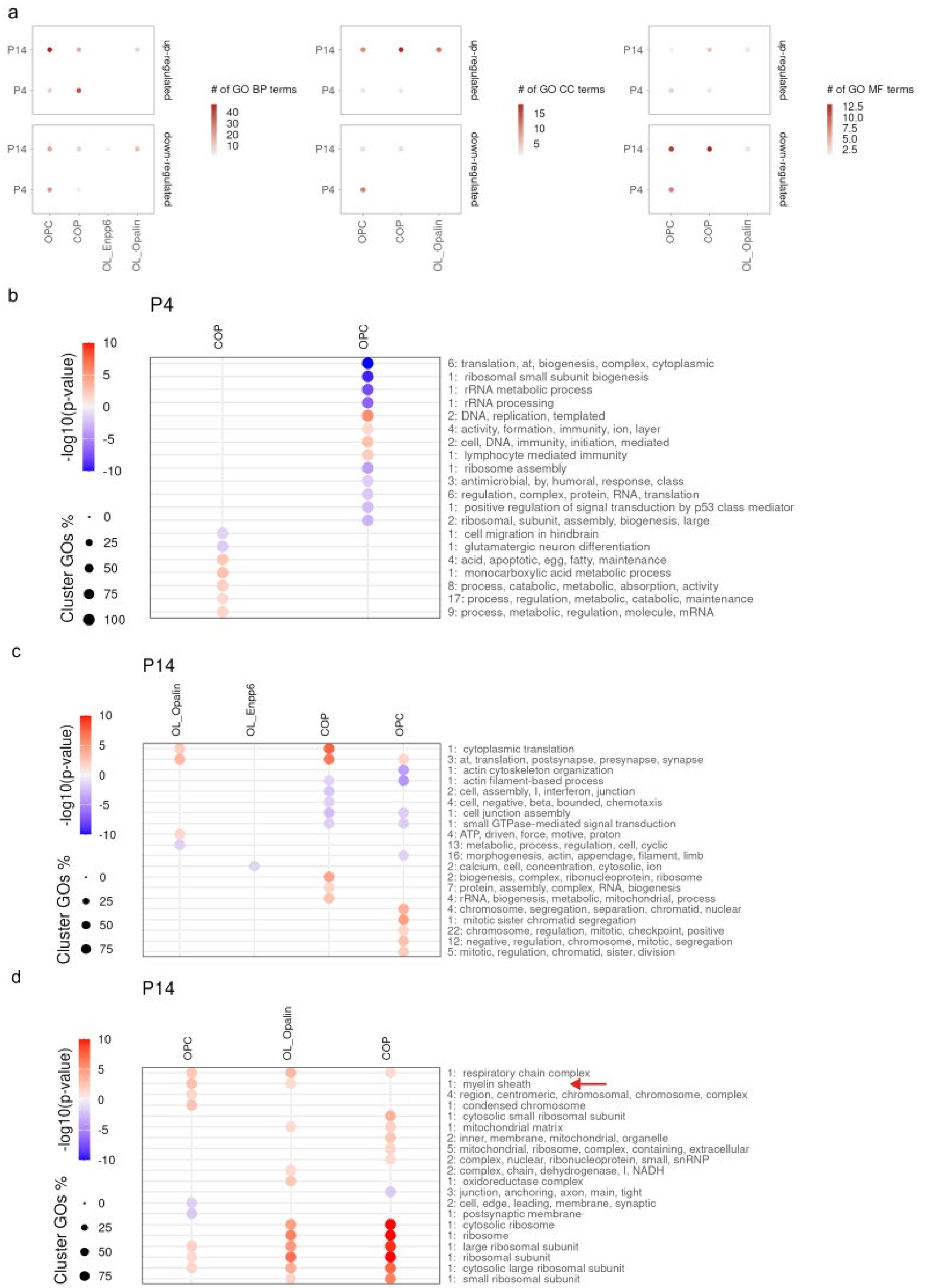

Supplementary Figure 8: **GO Biological Process and Cellular Component signatures in oligodendrocyte-lineage subtypes across development in *Scn2a*$^{p.A263V/+}$ mutant mice**

a: left: Number of significantly enriched GO Biological Process (BP) terms (Benjamini-Hochberg-adjusted *P* value < 0.05) among the top up- and down-regulated genes (*Scn2a*$^{p.\mathrm{A263V/+}}$ mutant versus control) in each oligodendrocyte-lineage cell subtype at P4 and P14, colored by the number of significantly enriched GO terms.

middle: The numbers of significantly enriched GO Cellular Component (CC) terms (Benjamini-Hochberg-adjusted *P* value < 0.05) among the top up- and down-regulated genes (*Scn2a*$^{p.\mathrm{A263V/+}}$ mutant versus control) in each oligodendrocyte-lineage cell subtype at P4 and P14, colored by the number of significantly enriched GO terms.

right: The numbers of significantly enriched GO Molecular Function (MF) terms (Benjamini-Hochberg-adjusted *P* value < 0.05) among the top up- and down-regulated genes (*Scn2a*$^{p.\mathrm{A263V/+}}$ mutant versus control) in each oligodendrocyte-lineage cell subtype at P4 and P14, colored by the number of significantly enriched GO terms.

b: GSEA of GO BP terms across oligodendrocyte-lineage cell subtypes at P4 comparing control and *Scn2a*$^{p.\mathrm{A263V/+}}$ mutant mice. Rows correspond to clusters of semantically related GO terms, obtained by grouping terms with overlapping enriched-gene sets and similar cell-type patterns. Dot color shows the mean signed −log10(*P*) for each cluster in a given cell type (positive = mutant-enriched; negative = control-enriched), truncated at ±10 to convey direction and relative magnitude (capped values are not exact *P*). Dot size indicates the fraction of terms in the cluster enriched in that cell type. Row labels give the number of terms per cluster followed by the five most frequent keywords. Top 20 clusters shown.

c: GSEA of GO BP terms across oligodendrocyte-lineage cell subtypes at P14 comparing control and *Scn2a*$^{p.\mathrm{A263V/+}}$ mutant mice. Rows correspond to clusters of semantically related GO terms, obtained by grouping terms with overlapping enriched-gene sets and similar cell-type patterns. Dot color shows the mean signed −log10(*P*) for each cluster in a given cell type (positive = mutant-enriched; negative = control-enriched), truncated at ±10 to convey direction and relative magnitude (capped values are not exact *P*). Dot size indicates the fraction of terms in the cluster enriched in that cell type. Row labels give the number of terms per cluster followed by the five most frequent keywords. Top 20 clusters shown.

d: GSEA of GO CC terms across oligodendrocyte-lineage cell subtypes at P14 comparing control and *Scn2a*$^{p.\mathrm{A263V/+}}$ mutant mice. Rows correspond to clusters of semantically related GO terms, obtained by grouping terms with overlapping enriched-gene sets and similar cell-type patterns. Dot color shows the mean signed −log10(*P*) for each cluster in a given cell type (positive = mutant-enriched; negative = control-enriched), truncated at ±10 to convey direction and relative magnitude (capped

values are not exact *P*). Dot size indicates the fraction of terms in the cluster enriched in that cell type. Row labels give the number of terms per cluster followed by the five most frequent keywords. Top 20 clusters shown.

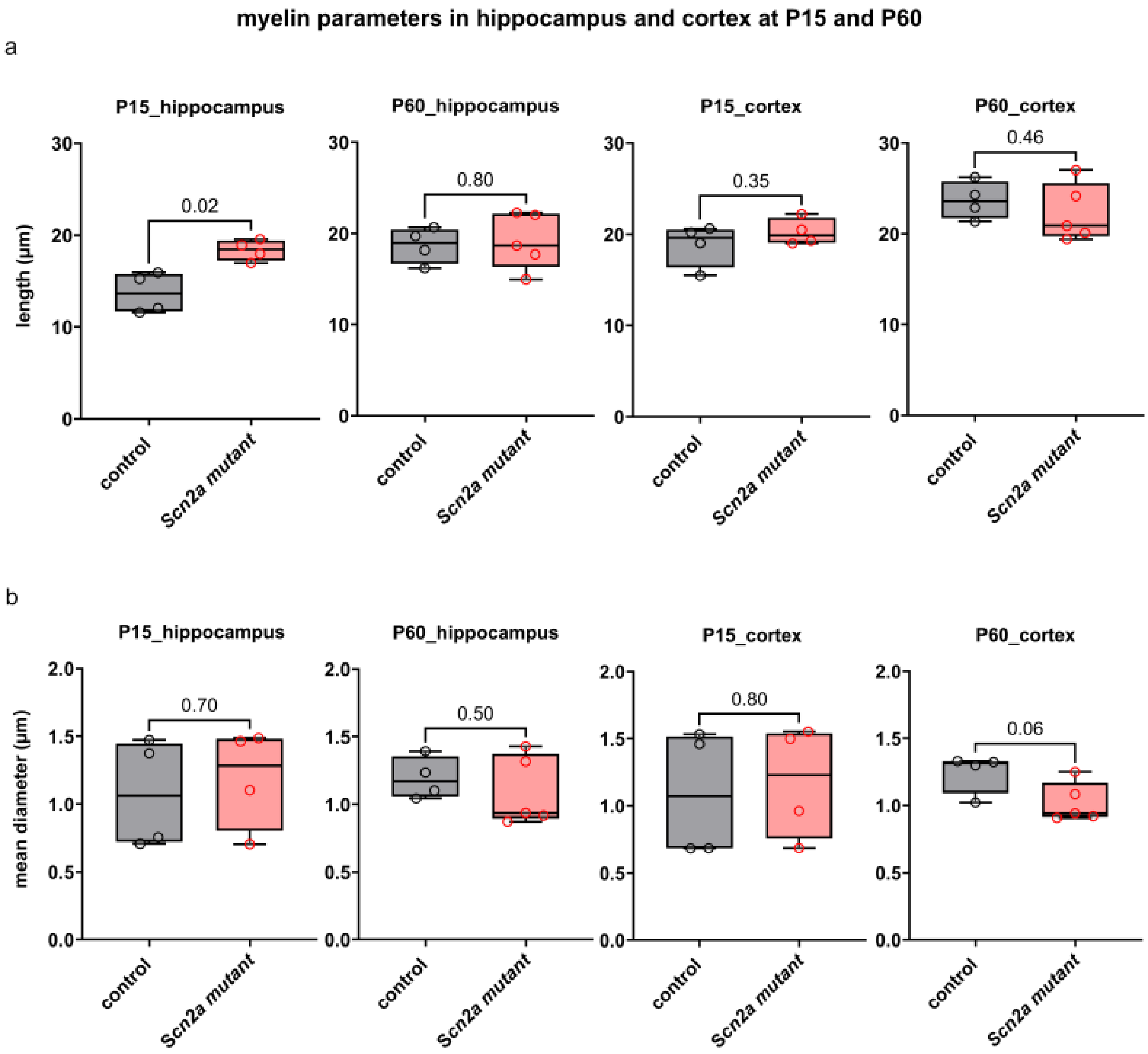

Supplementary Figure 9: Myelin parameters in hippocampus and cortex in *Scn2a*$^{+/+}$ control and *Scn2a*$^{p.A263V/+}$ mutant mice in different age groups

a: Myelin sheath length was assessed in hippocampus and cortex at P15 and P60. Average values per mouse are shown. In the cortex, layer V/VI was analyzed for P15, because at that age there is no myelin in layer I/II. At P60, layer I/II was analyzed, as layer V/VI was becoming too crowded to distinguish between single sheaths with our method.

P15 hippocampus: N=4 per group, Normality was assessed with Shapiro-Wilk test, comparisons between genotypes were made with Welch's t-test (p=0.02, t=3.77, df=4.47). Per mouse, 3 images were analyzed.

P60 hippocampus: N=4 for control and n=5 for *Scn2a*$^{p.A263V/+}$ mutant mice. Normality was assessed with Shapiro-Wilk test, comparisons between genotypes were made with Welch's t-test (p=0.80, t=0.26, df=6.77). Per mouse, 3 images were analyzed.

P15 cortex: N=4 per group, Normality was assessed with Shapiro-Wilk test, comparisons between genotypes were made with Welch's t-test (p=0.35, t=1.03, df=5.05). Per mouse, 3 images were analyzed.

P60 cortex: N=4 for control and n=5 for *Scn2a*$^{p.\mathrm{A263V/+}}$ mutant mice. Normality was assessed with Shapiro-Wilk test, comparisons between genotypes were made with Welch's t-test (p=0.46, t=0.77, df=6.81). Per mouse, 3 images were analyzed.

b: Myelin sheath mean diameter was assessed in hippocampus and cortex at P15 and P60. Average values per mouse are shown. In the cortex, layer V/VI was analyzed for P15, because at that age there is no myelin in layer I/II. At P60, layer I/II was analyzed, as layer V/VI was becoming too crowded to distinguish between single sheaths with our method.

P15 hippocampus: N=4 per group, Normality was assessed with Shapiro-Wilk test, comparisons between genotypes were made with Welch's t-test (p=0.70, t=0.41, df=5.95). Per mouse, 3 images were analyzed.

P60 hippocampus: N=4 for control and n=5 for *Scn2a*$^{p.\mathrm{A263V/+}}$ mutant mice. Normality was assessed with Shapiro-Wilk test, comparisons between genotypes were made with Welch's t-test (p=0.50, t=0.72, df=6.62). Per mouse, 3 images were analyzed.

P15 cortex: N=4 per group, Normality was assessed with Shapiro-Wilk test, comparisons between genotypes were made with Welch's t-test (p=0.80, t=0.26, df=5.93). Per mouse, 3 images were analyzed.

P60 cortex: N=4 for control and n=5 for *Scn2a*$^{p.\mathrm{A263V/+}}$ mutant mice. Normality was assessed with Shapiro-Wilk test, comparisons between genotypes were made with Welch's t-test (p=0.06, t=2.25, df=6.53). Per mouse, 3 images were analyzed.
